# Ribosomal S6 kinase 2-forkhead box protein O4 signaling pathway plays an essential role in melanogenesis

**DOI:** 10.1038/s41598-024-60165-9

**Published:** 2024-04-24

**Authors:** Dohyun Jeung, Ga-Eun Lee, Weidong Chen, Jiin Byun, Soo-Bin Nam, You-Min Park, Hye Suk Lee, Han Chang Kang, Joo Young Lee, Kwang Dong Kim, Young-Soo Hong, Cheol-Jung Lee, Dae Joon Kim, Yong-Yeon Cho

**Affiliations:** 1 BK21-4Th Team, College of Pharmacy, The Catholic University of Korea, 43, Jibong-ro, Wonmi-gu, Bucheon-si, Gyeonggi-do 14662 South Korea; 2 Biopharmaceutical research center, Ochang Institute of Biological and Environmental Science, Korea Basic Science Institute, 162, Yeongudanji-ro, Ochang-eup, Cheongwon-gu, Cheongju-si, 28119 Republic of Korea; 3 College of Pharmacy, The Catholic University of Korea, 43, Jibong-ro, Wonmi-gu, Bucheon-si, Gyeonggi-do 14662 South Korea; 4 Division of Life Sciences, Gyeongsang National University, 501, Jinju-daero, Jinju-si, Gyeongsangnam-do 52828 South Korea; 5 Anticancer Agent Research Center, Korea Research Institute of Bioscience and Biotechnology, 30, Yeongudanji-ro, Ochang-eup, Cheongju-si, Chongbuk 28116 South Korea; 6 Department of Immunology and Microbiology, School of Medicine, University of Texas Rio Grande Valley, MBMRF, 1.410, 5300, North L St., McAllen, TX 78504 USA

**Keywords:** RSK2, FOXO4, FOXO4 activity, Melanogenesis, Signaling pathway, Biochemistry, Physiology

## Abstract

Although previous studies have examined the signaling pathway involved in melanogenesis through which ultraviolet (UV) or α-melanocyte-stimulating hormones (α-MSH) stimuli act as key inducers to produce melanin at the stratum basal layer of the epidermis, the signaling pathway regulating melanogenesis is still controversial. This study reports that α-MSH, not UVA and UVB, acted as a major stimulus of melanogenesis in B16F10 melanoma cells. Signaling pathway analysis using gene knockdown technology and chemical inhibitors, the mitogen-activated protein kinase kinase (MEK)/extracellular signal-regulated kinase (ERK)/p90 ribosomal S6 kinase 2 (RSK2) played an important role in melanogenesis. Unexpectedly, LY294002, a PI3K inhibitor, increased melanogenesis without UV or α-MSH stimulation, suggesting that the PI3K/AKT signaling pathway may not be a major signaling pathway for melanogenesis. Chemical inhibition of the MEKs/ERKs/RSK2 signaling pathway using U0126 or BI-D1870 suppressed melanogenesis by stimulation of UVA or α-MSH stimulation, or both. In particular, the genetic depletion of RSK2 or constitutive active (CA)-RSK2 overexpression showed that RSK2 plays a key role in melanogenesis. Interestingly, forkhead box protein O4 (FOXO4) was phosphorylated by RSK2, resulting in the increase of FOXO4’s transactivation activity. Notably, the FOXO4 mutant harboring serine-to-alanine replacement at the phosphorylation sites totally abrogated the transactivation activity and reduced melanin production, indicating that RSK2-mediated FOXO4 activity plays a key role in melanogenesis. Furthermore, kaempferol, a flavonoid inhibiting the RSK2 activity, suppressed melanogenesis. In addition, FOXO4-wt overexpression showed that FOXO4 enhance melanin synthesis. Overall, the RSK2-FOXO4 signaling pathway plays a key role in modulating melanogenesis.

## Introduction

Melanin, a metabolic product of tyrosine, has many different roles, including pigment formation, free radical scavenging, and photoprotection^1–3^. Melanin protects skin from ultraviolet (UV)-induced DNA damage by the absorption of UV light^4^ and removes free radical to prevent oxidative stress-induced damage. In contrast, UV-reacted melanin, particularly pheomelanin, generates hydrogen peroxide and superoxide anion radicals, leading to DNA damage and DNA mutations^5,6^. Solar UV in the atmosphere is composed of 90–95% UVA and only 5–10% UVB^7,8^; UVB absorbed directly by DNA causes DNA structural damage, which evokes mutations and skin cancer development^8–10^.

Melanin synthesis is triggered by the binding of α-melanocyte stimulating hormone (α-MSH) to melanocortin 1 receptor (MC1R)^11,12^, located at the cytoplasmic membrane of melanocytes. When keratinocytes are exposed to UV, the synthesized POMC is cleaved and forms different hormones, including melanocyte-stimulating hormones (α-, β-, and γ-MSH), adrenocorticotropic hormone (CLIP), and β-various hormones, such as fat-stimulating hormone (β-EP)^11,13^. These hormones are processed and stored in immature secretory granules (ISGs) via maturation at the Golgi and secreted outside the cell^11^. The secreted melanin in vesicles (melanosome) is transported to keratinocyte. In melanogenesis, protein kinase A (PKA) activated by increased cyclic adenosine monophosphate (cAMP) plays a key signaling molecule in activating cAMP response element binding protein (CREB), which is a transcription factor to induce microphthalmia-associated transcription factor (MITF), is a key transcription factor that induces tyrosinase gene expression^12,14,15^. Thus, the signaling pathway has been targeted to manipulate melanogenesis.

Increased cAMP at the cytosol suppresses the PI3K/AKT signaling pathway, increasing melanogenesis by activating the glycogen synthase kinase-3β (GSK3β) signaling pathway^16^. Accumulating results have suggested that the PI3K/AKT signaling pathway might affect in melanogenesis differentially. For example, forkhead box protein O3 (FOXO3) and FOXO6, which are substrates of AKT, suppress melanogenesis via unknown mechanisms^17,18^. In contrast to FOXO 3 and 6, the FOXO1 signaling pathway induces melanogenesis^19^ by the induction of ER-Golgi vesicle trafficking, maturation, and transportation to keratinocytes^20^. Therefore, because it is still controversial for the role of melanogenesis, detailed signaling and molecular mechanisms for melanogenesis are necessary.

P90 ribosomal S6 kinase 2 (referred to as RSK2) is a serine/threonine protein kinase of the p90RSK protein family consisting of RSK1-4 and mitogen- and stress-activated kinase (MSK) 1–2^21^. This laboratory has studied the molecular mechanisms of RSK2 signaling involved in diverse cellular processes, including carcinogenesis, cancer progression, and cancer chemoresistance^22^. Accumulating literature shows that many transcription factors, including protein c-FOS, activating transcription factor 1 (ATF1), and CREB2, are newly identified as RSK2 substrates^22,23^. Moreover, RSK2 phosphorylates the RxRxxS/T or RxxS/T conserved target motifs of the substrates^24^, which is also phosphorylated by AKT^25,26^, suggesting that RSK2 may be involved in melanogenesis. Furthermore, RSK2 may involve in melanogenesis^25,27^ because RSK2 regulates the CREB2 and GSK3β activities^22,25^. On the other hand, no evidence has been reported yet to demonstrate the role of RSK2 in melanogenesis.

This study found that α-MSH/UVA-mediated RSK2 activity played an essential role in melanogenesis. In the signaling pathway, we found that RSK2 phosphorylated FOXO4 at Ser197 and Ser262, which are located at the DNA binding domain (DBD) and between the nuclear localization signal (NLS) and nuclear export signal (NES), respectively. Importantly, the phosphorylation of FOXO4 by RSK2 increased the transactivation activity of FOXO4, resulting in the induction of melanogenesis.

## Results

### Involvement of AKT and ERK signaling pathways in α-MSH-induced melanogenesis

**Figure 1:**
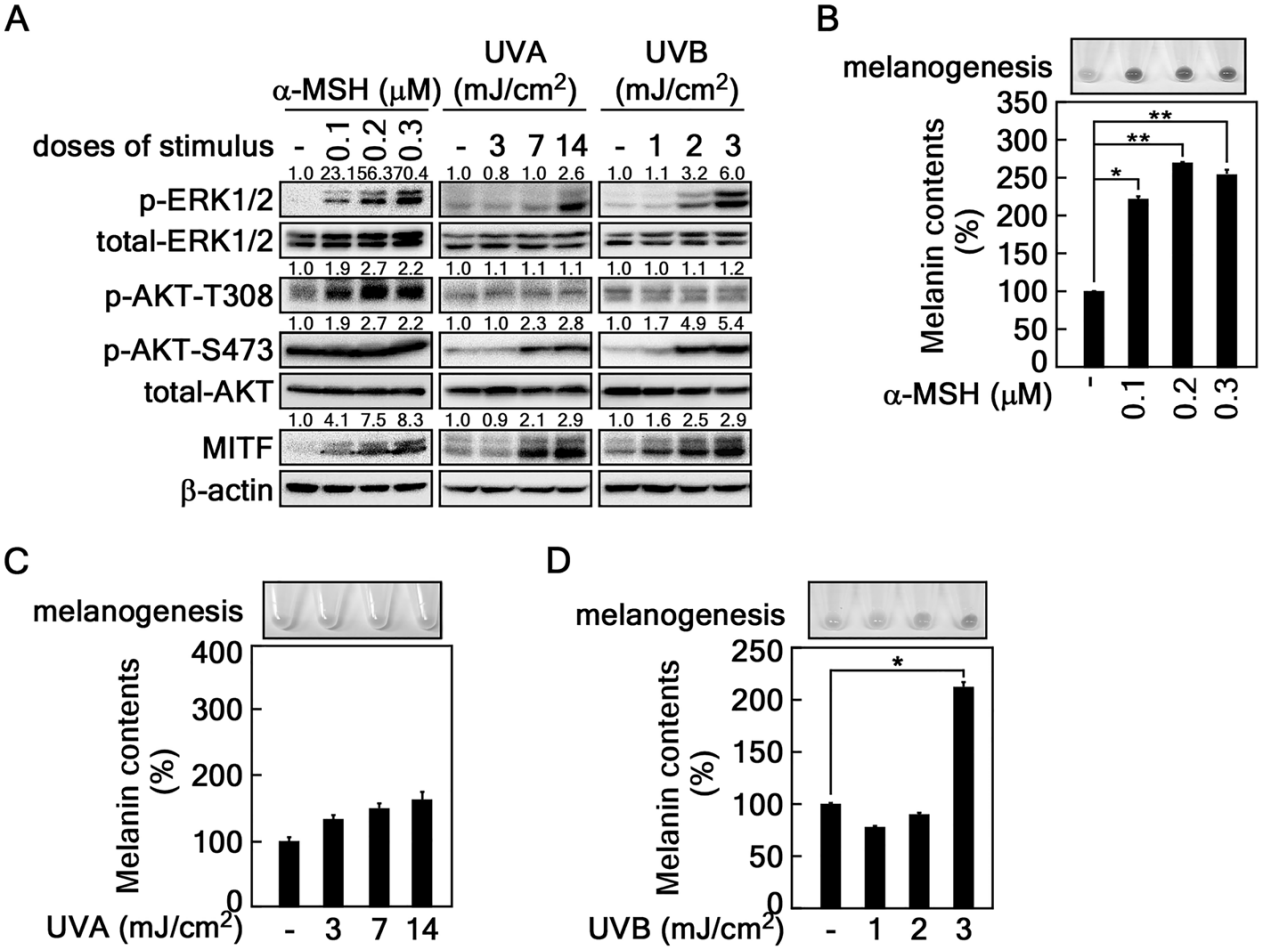
Involvement of AKT and ERK signaling pathways in α-MSH-induced melanogenesis. (**A**) Phosphorylation of ERK and AKT by α-MSH, UVA, or UVB stimulation in B16F10 melanoma cells. B16F10 cells (4.3 × 10^5^ cells/dish) were seeded in 60-mm dishes, stimulated with α-MSH (final concentration 100–300 nM), UVA (3, 7, 14 mJ/cm^2^), or UVB (1–3 mJ/cm^2^). The cells maintained for 36 h in complete growth medium after stimulation were harvested and the cell lysates were extracted using RIPA extraction buffer. The cell lysates (20 µg) were used to determine the indicated protein levels by Western blotting. β-actin was used as an internal control to verify the equal protein loading. (**B**–**D)** Determination of B16F10 cell darkness and melanin content. B16F10 melanoma cells seeded and stimulated as described in (**A**) were harvested. The effect of stimulation on the color change by melanin syntheis in the indicated doses of α-MSH (**B**), UVA (**C**), or UVB (**D**) was observed by photography using the cell pellets. The cell pellets were used to determine the melanin content as described in Materials and Methods. (**B**–**D**) Error bars indicate SEM. *p < 0.05, **p < 0.001 (Student *t*-test).

UV induces melanin synthesis in the skin by stimulation of keratinocytes to produce α-MSH or directly stimulating melanocytes^7,28^. Therefore, it is important to clarify whether stimulation of α-MSH or UVA/B or both triggers melanogenesis in melnaocytes. Moreover, because the depth of UV penetration is generally accepted with a range 20–60 μm skin thickness of 290–330 nm UV spectra^7^ and the skin stratum is about 3 μm thickness, UV irradiation may affect the melanogenesis in melanocytes of the skin. This study analyzed phosphorylation profiles of the well-known proteins involved in melanogenesis by stimulation of α-MSH, UVA, or UVB in B16F10 mouse melanoma cells. Interestingly, the phosphorylation profiles of the signaling molecules indicated that the protein levels of p-ERK1/2 and MITF, a transcription factor that regulates tyrosinase expression^29^, were increased by α-MSH, UVA, and UVB stimulation (Fig. 1A). Interestingly, α-MSH increased p-AKT-T308 phosphorylation, but not p-AKT-S473 phosphorylation, while, UVA and UVB increased p-AKT-S473 phosphorylation, but not p-AKT-T308 phosphorylation (Fig. 1A). However, total protein levels of ERK1/2 and AKT were not significantly changed by the α-MSH, UVA, or UVB in B16F10 mouse melanoma cells (Fig. 1A). These results suggested that the combinatorial regulation of p-ERKs/MITF and p-AKT-S473/MITF signaling pathways might play an essenstial role in melanogenesis depending on the different stimuli. To evaluate the direct effects of α-MSH, UVA, and UVB on the melanogenesis, melanin accumulation by B16F10 darkness and melanin content were measured. We found that α-MSH stimulation dramatically induced darkening and melanogenesis in B16F10 cells in a dose-dependent manner (Fig. 1B). However, either UVA or UVB marginally increased the B16F10 darkening and melanogenesis compared to α-MSH (Fig. 1C,D). These results suggest that α-MSH is a main extrinsic factor in inducing melanogenesis via the ERK and AKT signaling pathways, while alone of either UVA or UVB is not acted as a direct stimulus, but an additive role, in melanogenesis.

### Existence detouring pathway in α-MSH-induced melanogenesis

**Figure 2:**
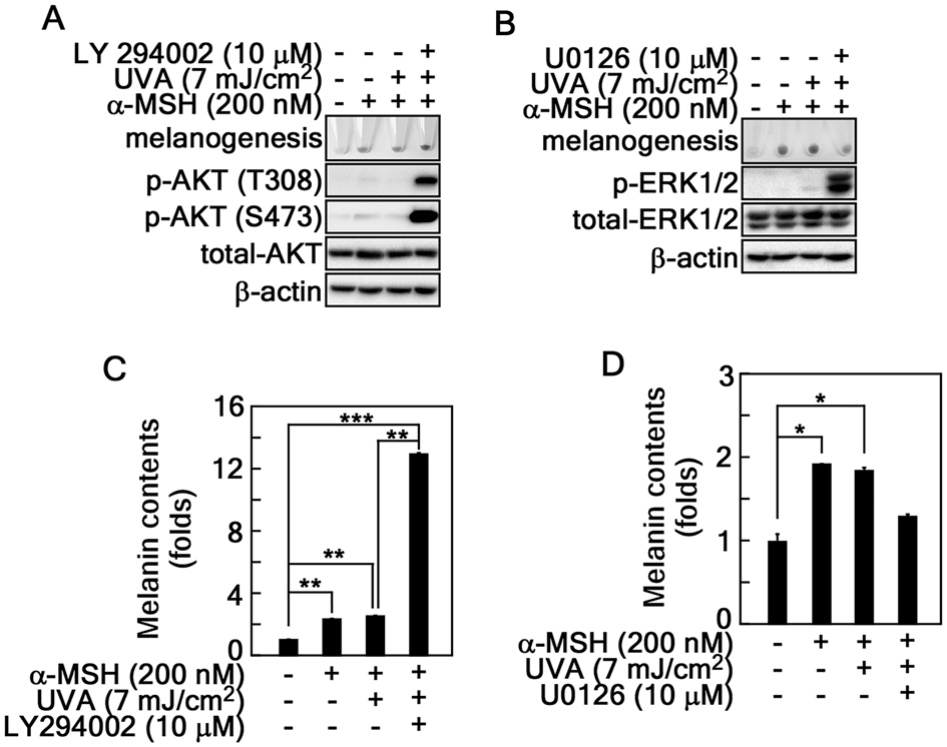
α-MSH-induced melanogenesis was detoured PI3K/AKT and MEK/ERK signaling pathways in B16F10 melanoma cells. (**A**,**B**) Determination of AKT and ERKs phosphorylation in melanogenesis. B16F10 cells (4.3 × 10^5^ cells/dish) were seeded in 60-mm dishes, stimulated with combination of α-MSH (200 nM), UVA (7 mJ/cm^2^), and LY294002 (10 µM) for (**A**) or U0126 (10 µM) for (**B**) as indicated, harvested, and melanin accumulation was determined by the pellet darkness. The cell lysates (20–30 µg) from the harvested cells were then used to evaluate the total- and phospho-protein levels by Western blotting using specific antibodies for PI3K/AKT signaling pathway (**A**) and MEK/ERK signaling pathway (**B**) as indicated. β-actin was used as an internal control to verify the equal protein loading. (**C**,**D**) Effect of PI3K-AKT or MEK-ERK signaling pathways in melanin content. The same set of B16F10 as shown in A and B were analyzed to determine the melanin content as described in Materials and Methods. Error bars indicate SEM. *p < 0.05, **p < 0.001, ***p < 0.0001 (Student *t*-test).

Although previous results have indicated that the AKT and ERK signaling pathways are acted as an essential signaling pathways in melanogenesis^28^, up to now the signaling pathways, mechanisms, and drivers to induce melanogenesis are controversial and unclear. Based on this rationale, the correlation of B16F10 cell darkness and either of AKT phosphorylation at Thr308 and Ser473 or ERK phosphorylation was determined using the signaling pathway inhibitors, such as LY294002, a PI3K inhibitor, and U0126, a MEK inhibitor, respectively (Fig. 2A,B). Surprising results were obtained that blocking the PI3K-AKT signaling pathway by a treatment of LY294002 strongly induced B16F10 melanoma cell darkness (Fig. 2A). At the same time, AKT phosphorylation at Thr308 and Ser473 was increased by a co-treatment of α-MSH/UVA (Fig. 2A) in contrast to our expectation. Moreover, although U0126 was treated, ERK1/2 phosphorylation levels were dramatically increased by α-MSH/UVA, melanoma cell darkness was not enhanced as similar as shown in α-MSH and α-MSH/UVA stimulation (Fig. 2B). Notably, melanin content measurement showed that the inhibition of PI3K-AKT signaling pathway by LY294002 treatment increased the melanogenesis with synergistical effect to α-MSH and α-MSH/UVA stimulation (Fig. 2C) and the inhibition of MEK-ERK signaling pathway by U0126 treatment abolished melanogenesis with inverse effect to α-MSH and α-MSH/UVA stimulation (Fig. 2D). These results showed that there are detour signaling pathways to bypass ERKs and AKTs in the melanogenesis process.

### Critical role of RSK2-mediated signaling pathway in α-MSH-induced melanogenesis

**Figure 3:**
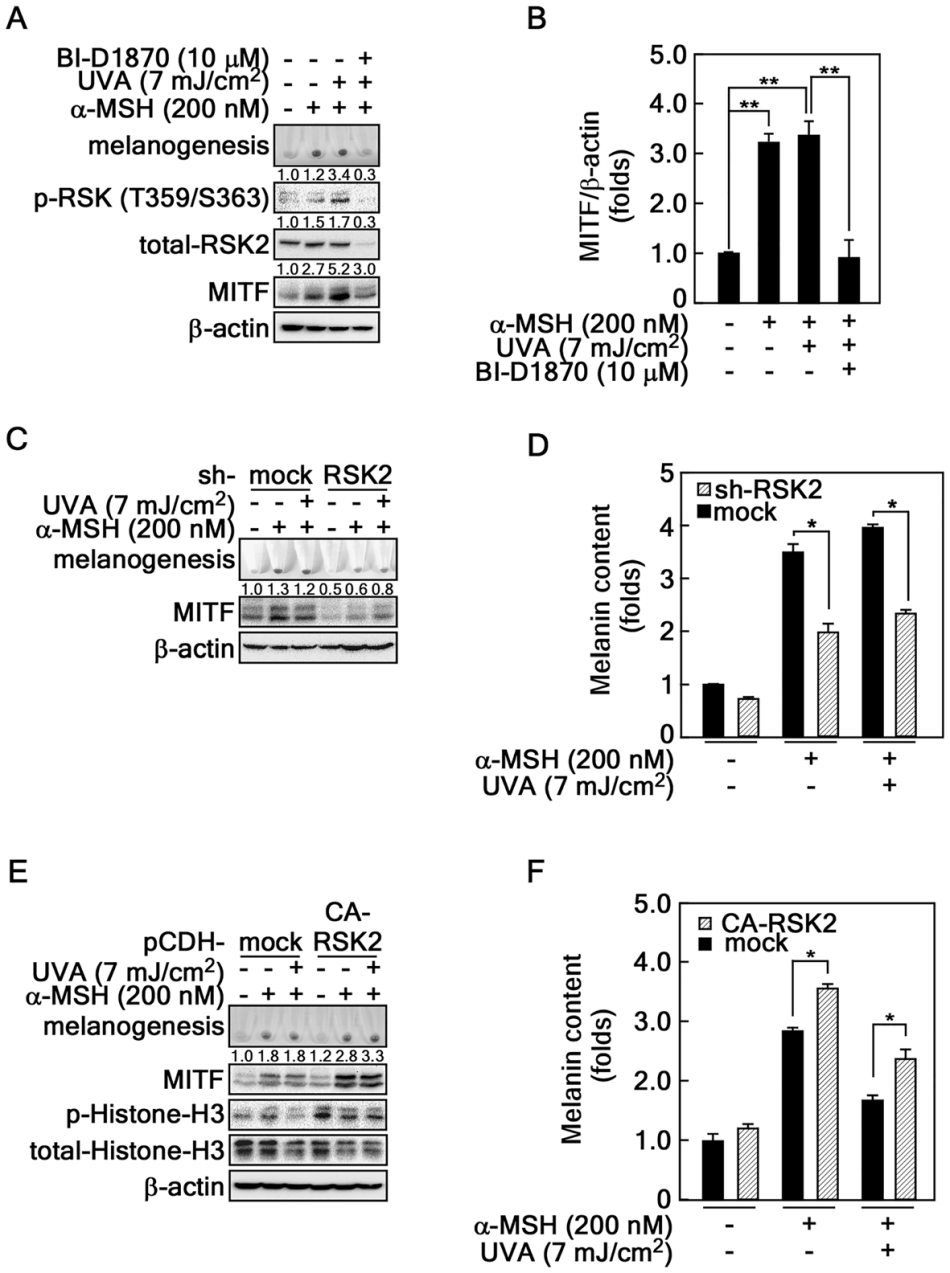
Roles of RSK2 in α-MSH-induced melanogenesis. (**A**,**B**) Chemical inhibition of RSK2 activity suppressed melanogenesis. RSK2 inhibition abrogates α-MSH-induced melanogenesis. B16F10 cells (4.3 × 10^5^ cells/dish) were treated with a combination of α-MSH, UVA, and BI-D1870 for 36 h as indicated and harvested by centrifugation. The accumulated melanin was visualized by photography of the pellets. The cell lysates from the pellets were prepared to determine the phospho-RSK-T359/S363 and total-RSK2 and -MITF protein levels by Western blotting using specific antibodies as indicated (**A**). The MITF protein level changes were graphed by measuring the intensities of MITF and β-actin using Image J software (**B**). (**C**,**D**) RSK2 knockdown abrogates α-MSH-induced melanogenesis. B16F10 cells stably expressing sh-mock or -RSK2 were treated with the combination of α-MSH and UVA for 36 h as indicated and harvested by centrifugation. The accumulated melanin was visualized by photography of the pellets. The cell lysates from the pellets were prepared to determine the total-MITF protein levels by Western blotting using specific antibodies as indicated (**C**). Half of the cell pellets in **C** were subjected to measure the melanin content assay as described in “Materials and Methods”. The fold changes in melanin content were graphed (**D**). (**E**,**F**) Overexpression of constitutive active (CA)-RSK2 increases melanogenesis. B16F10 melanoma cells stably expressing mock or CA-RSK2 were treated with the combination of α-MSH and UVA for 36 h as indicated and harvested by centrifugation. The accumulated melanin was visualized by photography of the pellets. The cell lysates from the pellets were prepared to determine the phospho-histone H3 and total-MITF and -histone H3 protein levels by Western blotting using specific antibodies as indicated (**E**). Half of the cell pellets in **E** were subjected to measure the melanin content assay as described in “Materials and Methods”. The fold changes in melanin content were graphed (**F**). (**A**,**C**,**E**) β-actin was used as an internal control to verify the equal protein loading. (**B**,**D**,**F**) Error bars indicate SEM. *p < 0.05, **p < 0.001 (Student *t*-test).

Previous our reports indicated that RSK2 acted as a signaling node to detouring ERKs when the cells were stimulated with basic fibroblast growth factor^30,31^. Moreover, RSK2 played an essential role in the proliferation of chemoresistant cancer cells depending on the different stimuli^31–33^. These results suggested that RSK2 influenced melanogenesis induced by α-MSH/UVA. When the cells were treated with BI-D1870, an inhibitor of RSK2 C-terminal kinase, in B16F10 melanoma cells, the cell darkness was strongly inhibited compared to co-treatment of α-MSH alone and α-MSH/UVA (Fig. 3A). Additionally, BI-D1870 treatment reduced the phosphorylation of RSK at Ser359/S363 and total RSK2 levels (Fig. 3A). Kaempferol, a natural compound specifically inhibiting RSK2 activity^32,34^, reduced RSK2 protein levels, while the reduction rate was smaller than BI-D1870 (Supplementary Fig. 1). In particular, the MITF protein levels were correlated with B16F10 cell darkness (Fig. 3A,B). Moreover, the stable depletion of RSK2 using pLenti-sh-RSK2 also suppressed the α-MSH-induced cell darkness (Fig. 3C). The suppressed cell darkness was caused by the inhibition of melanogenesis in B16F10 cell (Fig. 3D). In contrast, the acceleration ratio of the B16F10 cell darkeness was observed by overexpression of constitutive active (CA)-RSK2 when the cells were stimulated with α-MSH (Fig. 3E). This increase in CA-RSK2 overexpression was correlated with the melanin content in B16F10 melanoma cell (Fig. 3F). These results showed that RSK2 participated in α-MSH/UVA-induced melanogenesis in B16F10 melanoma cells.

### Wiring of RSK2-FOXOs interaction

**Figure 4:**
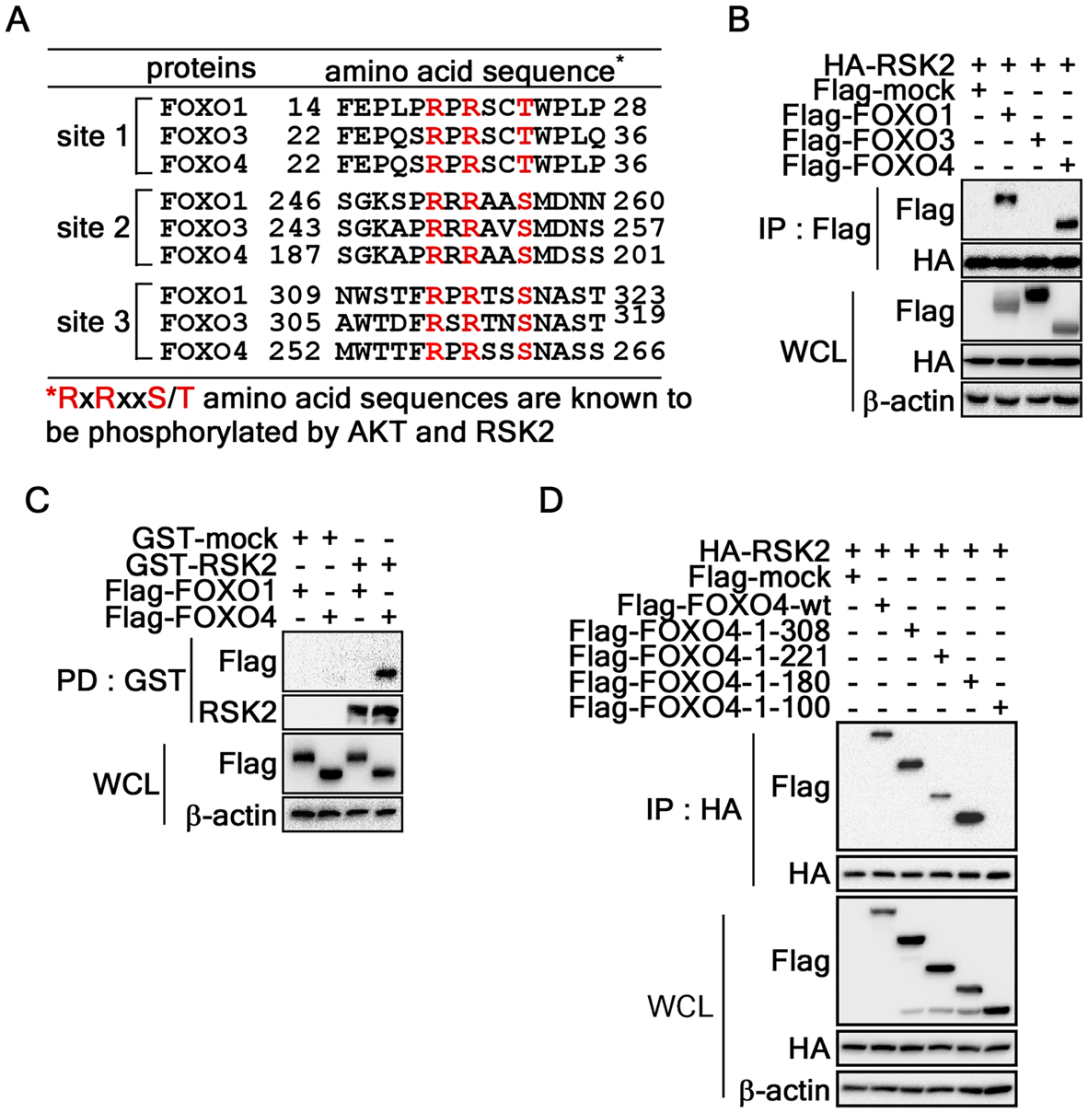
FOXO4 is a novel RSK2 binding partner. (**A**) Identification of AKT and RSK2 target phospho-consensus sequences in FOXO 1, 3, and 4. Amino acid sequences of FOXO 1, 3, and 4 were used to identify the AKT and RSK2 target phospho-consensus sequences, RxRxxS/T or RxxS/T. (**B**) RSK2 interacts with FOXO1 and FOXO4. HEK293T cell lysates (200 µg) transiently expressing HA-RSK2 and each of Flag-FOXO 1, 3, and 4 were used to evaluate the interaction by IP and Western blotting using specific antibodies as indicated. (**C**) Confirmation of RSK2 and FOXOs interaction by GST-RSK2 pulldown assay. GST-RSK2 expressed in E. coli bound to GST-bead were used to evaluate the interaction of FOXO1 or 4 by an ex vivo pull-down assay and Western blotting as indicated. (**D**) Identification of the FOXO4 binding domain with RSK2. The cell lysates (200 µg) transiently expressing HA-RSK2 and each of Flag-FOXO4-wt and -serial deletion mutants (Supplementary Fig. 2C) in HEK293T cells were used to determine the binding domain by IP and Western blotting using specific antibodies as indicated. (**B**–**D**) β-actin was used as an internal control to verify the equal protein loading.

RSK2, a member of the serine/threonine kinase superfamily, is activated in response to stimuli such as growth factors, peptide hormones, and UV^22,33^. Previous studies showed that UV-induced activated RSK2 localizes to the nucleus and phosphorylates diverse substrates, including CREB2 and histone H3^22^. Recently, we demonstrated that noncanoical wiring of the AKT and RSK2 signaling pathways plays a critical role in the G_1_/S cell cycle transition^24^. Based on the rationale that the AKT-FOXO axis is a canonical signaling pathway that regulates melanogenesis, and RSK2 has been classified as an AGC kinase family member, similar to AKT^25,35^, we hypothesized and examined the possibility that RSK2 might play an important role in melanogenesis. Since LY294002 increased the melanogenesis (Fig. 2A,C), we excluded the direct connection between AKT-FOXO signaling pathway. Moreover, FOXO phosphorylation at RxRxxS/T and RxxS/T, which are also classified as RSK2-mediated phosphorylation target motifs^36^, plays an important role in melanogenesis, we proposed that RSK2 may act as a FOXO kinase. Since FOXO6 is predominantly expressed in the brain tissue^37^, we compared the amino acid sequence FOXO1, FOXO3, and FOXO4 to confirm which one contained RxRxxS/T or RxxS/T motif(s) for RSK2 targeting. The 3 RxRxxS/T motifs (designated as site 1, 2, and 3, respectively) (Fig. 4A) were identified at the unstructured N-terminal, nuclear localization signal (NLS), and nuclear export signal (NES) (Supplementary Fig. 2A). The immunoprecipitation experiment to identify RSK2-targeted FOXO(s) showed that FOXO1 and FOXO4 interacted with RSK2 (Fig. 4B). Moreover, RSK2 interaction with FOXO4, not FOXO1, was confirmed by pull-down assay using a partially purified GST-RSK2 protein (Supplementary Fig. 2B) and cell lysates transiently expressing Flag-FOXO1 and -FOXO4, respectively (Fig. 4C). Importantly, the specific binding domain of FOXO4 with RSK2 was determined by IP using the cell lysates transiently expressing HA-RSK2 and each of the FOXO4 serial deletion mutants (Supplementary Fig. 2C). The IP results of RSK2 showed that a deletion of FOXO4 to 180 from C-terminal end showed an interaction with RSK2. On the other hand, the band using FOXO4-1-100 by the deletion of additional 80 amino acid residues (DNA binding domain, DBD) had totally disappeared (Fig. 4D). These results showed that RSK2 interacted with FOXO4 via DBD spanning aa 100-180.

### RSK2 phosphorylates FOXO4 at Ser197 and Ser262

**Figure 5:**
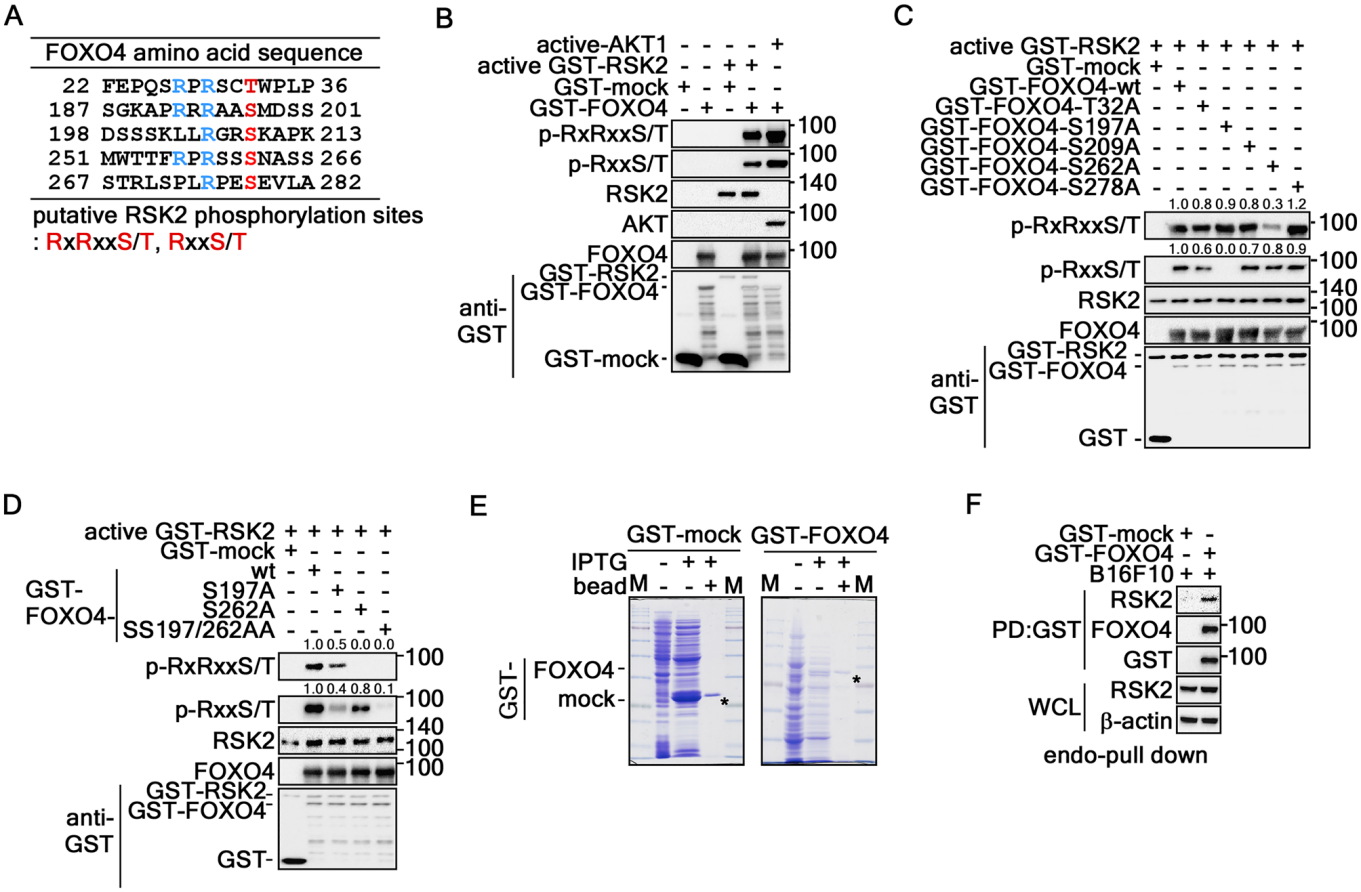
RSK2 phosphorylates FOXO4 at Ser197 and Ser262. (**A**) Prediction of the putative RSK2 phosphorylation sites in FOXO4. Detailed prediction of the FOXO4 aa sequence to identify the RSK2 phospho-target consensus motifs, RxRxxS/T and RxxS/T, were conducted, analyzed, and summarized. (**B**) RSK2 phosphorylates FOXO4. GST-FOXO4 expressed in E. coli bound to GST-bead was used to determine if FOXO4 could be phosphorylated by RSK2 by an in vitro kinase assay. The phosphorylation of FOXO4 by RSK2 was determined by Western blotting using p-RxRxxS/T or p-RxxS/T specific antibodies as indicated. Active AKT was used as a positive control. (**C**) RSK2 phosphorylates FOXO4 at Ser197 and Ser262. GST-FOXO4-wt and -point mutants (Supplementary Fig. 3B) expressed in E. coli and bound to GST-bead (Supplementary Fig. 3C) were used to determine the RSK2 phospho-target amino acids by an in vitro kinase assay as indicated. The p-RxRxxS/T- or -RxxS/T-specific antibodies were used to visualize the phosphorylated FOXO4 proteins. (**D**) Confirmation of RSK2-mediated FOXO4 phosphorylation at Ser197 and Ser262. GST-FOXO4-wt and -single or double point mutants expressed in E. coli and bound to GST-bead (Supplementary Fig. 3D) were used to confirm the RSK2 phospho-target amino acids by an in vitro kinase assay as indicated. The p-RxRxxS/T- or -RxxS/T-specific antibodies were used to visualize the phosphorylated FOXO4 proteins. (**E**,**F**) Ex vivo pull down assay to determine the GST-FOXO4 interaction with endogenous RSK2. GST-FOXO4 proteins conjugated with GST-sepharose beads (**E**) were used to determine the interaction with endogenous RSK2 in B16F10 cell lysate. FOXO4-bound RSK2 was visualized by Western blotting (**F**). β-actin was used as an internal control to verify the equal protein loading. * in (**E**) indicates GST and GST-FOXO4 as indicated.

RSK2 may act as a kinase of FOXO4 because RSK2 interacts with FOXO4 (Fig. 4). Thus, the amino acid sequence of FOXO4 was analyzed to identify the RxRxxS/T or RxxS/T phosphorylation target motifs. This study found five possible phosphorylation target motifs scattering in the N-terminal half of FOXO4, which harbors DBD, nuclear localization signal (NLS), and nuclear export signal (NES) (Fig. 5A). Based on the prediction result, FOXO4 was partially purified in E. coli (Supplementary Fig. 3A), and an in vitro kinase assay was conducted. RSK2-mediated phosphorylated FOXO4 bands were observed when phosphorylated FOXO4 was detected by western blotting using RxRxxS/T- or RxxS/T-specific antibody (Fig. 5B). A single point mutation was introduced at at Thr32, Ser197, Ser209, Ser262, or Ser278 in pGEX-5X-1 GST-fusion vector to determine the specific amino acids of FOXO4 phosphorylated by RSK2 (Supplementary Fig. 3B). The GST-FOXO4 proteins harboring each single point mutation were partially purified (Supplementary Fig. 3C), and used for the in vitro kinase assay using active RSK2. The results showed that the RSK2-mediated phosphorylated GST-FOXO4 band disappeared in GST-FOXO4-S262A by western blotting using the RxRxxS/T-specific antibody (Fig. 5C). Moreover, the RSK2-mediated phosphorylated FOXO4 band disappeared using RxxS/T-specific antibody according to western blotting when the GST-FOXO4-S197A protein was used as an RSK2 substrate for the in vitro kinase assay (Fig. 5C). These results strongly suggest that RSK2 may phosphorylate FOXO4 at Ser197 and Ser262. A double mutation was re-introduced in GST-FOXO4 at Ser197 and Ser262 (designated as FOXO4-SS197/262AA) and partially purified (Supplementary Fig. 3D) to clarify this suggestion. In particular, a double mutation of GST-FOXO4 at Ser197 and Ser262 (GST-FOXO4-SS197/262AA) abolished RSK2-mediated phosphorylation in both RxRxxS/T- and RxxS/T-specific antibody (Fig. 5D). The physiological interaction between FOXO4 and endogenous RSK2 was confirmed by ex vivo pull down assay using B16F10 cell lysates and GST-FOXO4 beads (Fig. 5E,F). These results demonstrated that FOXO4 is acted as an RSK2 substrate to be phosphorylated at Ser197 and Ser262.

### RSK2 regulates the transactivation activity of FOXO4

**Figure 6:**
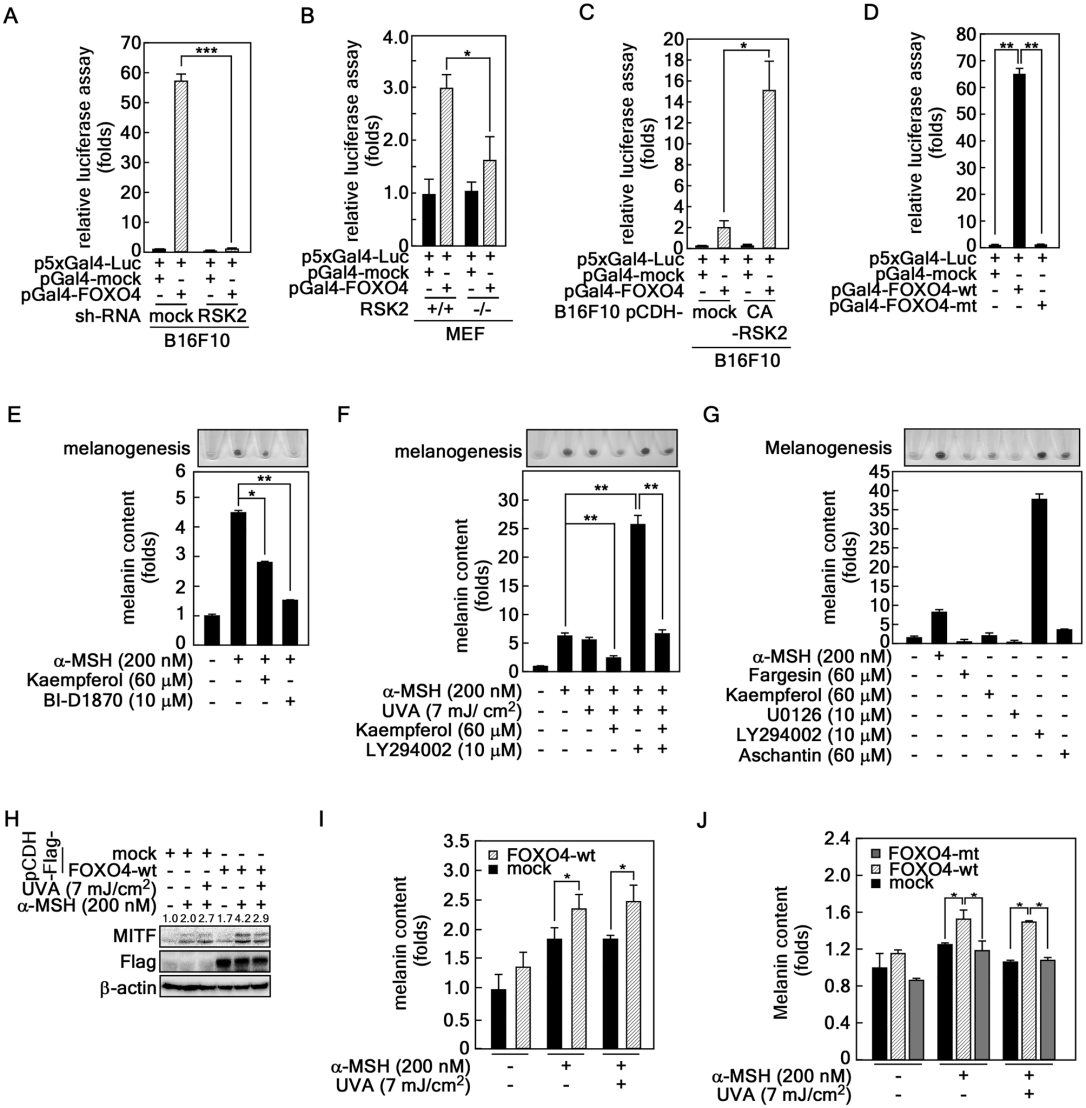
RSK2 enhances melanogenesis by upregulation of FOXO4 transactivation activity. (**A**) RSK2 knockdown suppresses FOXO4 transactivation activity. B16F10 cells stably expressing sh-mock or -RSK2 were established. The cells (3.8 × 10^4^ cells/well) were seeded into 12-well plates, transfected with *p5xGal4-luc* and *pGal4-mock* or *-FOXO4* as indicated, and cultured for 24 h. The cells were treated with α-MSH (200 nM) for 1 h before cell lysed using luciferase lysis buffer (Promega). The cell lysates (100 μl) were used to measure the firefly luciferase activity. Equal transfection efficiency was normalized to the *phRL-luc* reporter plasmid. (**B**) RSK2 knockdown abolished FOXO4 transactivation activity. RSK2^+/+^ and RSK2^−/−^ mouse embryonic fibroblasts (3 × 10^4^ cells/well) were seeded into 12-well plates, transfected with a combination of* p5xGal4-luc* and either *pGal4-mock* or *-FOXO4* as indicated, and cultured for 24 h. The firefly luciferase activity was evaluated as described in (**A**). (**C**) Overexpression of CA-RSK2 increases the FOXO4 transactivation activity. B16F10 cells (4.5 × 10^4^ cells/well) stably expressing mock or CA-RSK2 were seeded into 12-well plates, transfected with *p5xGal4-luc* and either *pGal4-mock* or *-FOXO4* as indicated, and cultured for 24 h. The cells were treated with α-MSH (200 nM) for 1 h before cell lysed using luciferase lysis buffer (Promega). The firefly luciferase activity was evaluated as described in (**A**). (**D**) The mutation of FOXO4 at Ser197/Ser262 abolished FOXO4 transactivation activity. B16F10 cells transiently co-transfected with *p5xGal4-Luc* and either *pFOXO4-wt* or *-mt* were in 12-well plates used to analyze the transactivation activity of FOXO4 by firefly luciferase assay. The equal transfection was compansated by *Renilla* luciferase activity using *phRL-SV40*. (**E**) RSK2 inhibition using chemical inhibitors suppressed melanin synthesis. B16F10 cells (4.3 × 10^5^ cells/dish) were treated with α-MSH and either kaempferol or BI-D1870 for 36 h, harvested, and photographed. The melanin content in the cells was measured as described in “Materials and Methods”. (**F**) Inhibitory effects of kaempferol on melanin synthesis. B16F10 cells (4.3 × 10^5^ cells/dish) were treated with α-MSH and combination of UVA, kaempferol, or LY294002 for 36 h, harvested, and photographed. The melanin content in the cells was measured as described in “Materials and Methods”. (**G**) Basal properties of chemical compounds on melanin synthesis in B16F10 cells. B16F10 cells (4.3 × 10^5^ cells/dish) were treated with the indicated dose of chemical compounds, including fargesin, kaempferol, U0126, LY294002, or aschantin for 36 h, harvested, and photographed. The melanin content in the cells was measured as described in “Materials and Methods”. α-MSH was used as a positive control. (**H**,**I**) Overexpression of FOXO4-wt increases MITF-mediated melanogenesis. MITF protein levels (**H**) and melanogenesis (**I**) were compared in B16F10 melanoma cells stably expressing mock or FOXO4-wt by α-MSH/UVA treatment. β-actin in (**H**) was used as an internal control to verify the equal protein loading. (**J**) RSK2-FOXO4 signaling pathway involves melanogenesis. B16F10 melanoma cells stably expressing mock, FOXO4-wt, or FOXO4-mt were stimulated with α-MSH or α-MSH/UVA and then compared melanin content. (**A**–**F**,**I**,**J**) Error bars indicate SEM. *p < 0.05, **p < 0.001 (Student *t*-test).

Since α-MSH-induced melanogenesis was regulated by RSK2 activity (Fig. 3) and RSK2 phosphorylated FOXO4 at Ser197 and Ser262 (Fig. 5), we hypothesized that RSK2-mediated FOXO4 phosphorylation might play an essential role in α-MSH-induced melanogenesis via FOXO4 transactivation activity. At first, we evaluated the effect of RSK2 knockdown in FOXO4 transactivation activity using the expression vectors, such as *pGal4-FOXO4* and *p5xGal4-luciferase* reporter plasmids^38^ (Supplementary Fig. 4A), in B16F10 melanoma cells. The FOXO4 transactivation activity in sh-mock cells was abolished by RSK2 knockdown (Fig. 6A), indicating that RSK2 regulates FOXO4 transactivation activity. Similarly, the decreased FOXO4 transactivation activity by RSK2 knockout (RSK2^−/−^) in MEFs was observed compared to RSK2 wildtype (RSK2^+/+^) MEFs (Fig. 6B). In contrast to knockdown or knockout of RSK2, overexpression of constitutive active RSK2 (CA-RSK2) enhanced the FOXO4 transactivation activity approximately 15 fold compared to that of mock expression in B16F10 mouse melanoma cells (Fig. 6C). Importantly, FOXO4-wt-mediated increased luciferase activity, approximately 65 folds, were totally abolished by a double mutation at Ser197 and Ser262 (Fig. 6D and Supplementary Fig. 4B), indicating that RSK2-mediated FOXO4 phosphorylation at Ser197 and Ser262 is critically important to regulate FOXO4 transactivation activity. These results further supported that FOXO4 transactivation activity is positively regulated by RSK2, resulting in an increase in melanogenesis. Accordingly, we examined the effects of RSK2 inhibitors, kaempferol and BI-D1870^39^, on melanogenesis. These compounds inhibited α-MSH-induced B16F10 cell darkening and melanin production (Fig. 6E). As expected, kaempferol strongly abrogated α-MSH/UVA-induced B16F10 darkening and melanogenesis (Fig. 6F). Moreover, kaempferol abolished LY294002-induced B16F10 darkening and melanogenesis (Fig. 6F), suggesting that RSK2 inhibitors might be applicable to skin whitening. Additionally, we further confirmed that fargesin, kaempferol and aschantin, which were natural compounds harboring chemopreventive activity of skin cancer development^34,40^, are not contained B16F10 darkening and melanogenesis, while LY294002 harbored the ability for B16F10 darkening and melanogenesis (Fig. 6G). To evaluate the effect of FOXO4 in melanogenesis, we confirmed that overexpression of FOXO4-wt increased MITF protein levels by α-MSH stimulation (Fig. 6H), resulting in increased of malanogenesis (Fig. 6I). Importantly, the increased melanogenesis by FOXO4-wt was suppressed by FOXO4-mt (Fig. 6J). However, since the difference of melanogenesis by FOXO4-wt overexpression, we concluded that RSK2-mediated melanogenesis is not only involved FOXO4 but also other unidentified factors. Overall, RSK2-mediated FOXO4 activity signaling pathway involves in α-MSH-induced melanogenesis.

## Discussion

Sunlight exposure of skin can trigger skin pigmentation by stimulating melanocytes^41^. In this process, keratinocytes stimulated by UV release α-MSH, which acts as a ligand of MC1R, a G-protein coupled receptor, at the cytoplasmic membrane of melanocytes^12^. The interaction between α-MSH and MC1R triggers melanin synthesis and increases melanosome formation. Although this metabolic pathway appears well adapted and accepted, the molecular mechanisms to regulate melanogenesis have not been completely elucidated. For example, α-MSH binding to MC1R induces the activation of adenylyl cyclase, resulting in an increase in the intracellular levels of cAMP and subsequently upregulating the TYR, tyrosinase-related protein 1 (TYRP1) and TYRP 2 protein levels. This process was mediated through which cAMP elevation predominately activates PKA, which phosphorylates and activates CREB, resulting in an increase of TYRP 1 and 2 gene expression^42^. Another signaling pathway to induce melanogenesis is the Ras-mediated MEK/ERK signaling pathway. In this process, c-receptor tyrosine kinase (KIT)-mediated ERK phosphorylation induces CREB phosphorylation, activating melanin synthesis via MITF stability regulation^28,43^. Recent results suggested that many of transcription factors, including activating transcription factor 1 (ATF1), CREB3, GSK3β, and ETS transcription factor ELK3 (ELK3), known as ERK substrates, are regulated by RSK2-mediated phosphorylation^22,25,34^. Therefore, RSK2 may play an important role in melanogenesis. This hypothesis is supported by melanogenesis inhibition via treatment with flavonoids, including isoorientin, catechin, coumaric acid, and kaempferol 7-*O*-glucuronide, derived from Gentiana^44^, Phyllostachys nigra, Cryptotaenia japonica^45^, and dried pomegranate concentrate powder^46^. On the other hand, the molecular targets for melanogenesis inhibition have not been elucidated. This study found that MEK/ERK signaling inhibition by the MEK inhibitor U0126 suppressed melanogenesis (Fig. 2B). Surprisingly, U0126 induced ERK phosphorylation in B16F10 melanoma cells by α-MSH and UVA stimulation (Fig. 2B). In contrast, LY294002, a PI3K inhibitor, dramatically increased melanogenesis by α-MSH and UVA stimulation (Fig. 2A). Surprisingly, AKT phosphorylation was increased at Thr308 and Ser473 (Fig. 2A). Hence, there are circumventing pathways to detour the PI3K/AKT and MEK/ERK signaling cascades. Importantly, BI-D1870, an RSK2 inhibitor, inhibited the pellet darkness and MITF protein levels (Fig. 3A), indicating that RSK2 might act as an upstream kinase that critically modulates melanogenesis. Moreover, the role of RSK2 as an upstream signaling molecule of melanogenesis was provided by the RSK2 depletion. The results suggested that RSK2 knockdown inhibited the α-MSH and UVA-induced MITF protein levels (Fig. 3B). These results strongly suggest that RSK2 plays a key role in melanogenesis.

BI-D1870, which was licensed by Boehringer Ingelheim Pharma GmbH & Co., is a specific cell-permeable inhibitor targeting RSKs including RSK1, RSK2, RSK3 and RSK4 with IC_50_ values of approximately 0.031, 0.024, 0.018 and 0.015 μM, respectively^47^. With the low IC_50_ values against RSKs, it also inhibited the activities of other kinases including MST2, GSK3β, MARK3, CK1, Aurora B and PLK1 with IC_50_ values of approximately 0.86, 1.59, 2.20, 0.45, 0.34 and 0.10 μM, respectively^47^. Previous results showed that kaempferol is a specific RSK2 inhibitor by targeting the active pocket of the N-terminal kinase domain^48^. Pyrrolopyrimidine (fmk), a fluoromethylketone molecule, specifically inhibits the RSK2 activity by targeting RSK2 CTKD with 15 nM of IC_50_ and 200 nM of the half-maximal effective concentration (EC_50_)^47^. A molecular mechanism study showed that fmk formed a covalent bond of chloromethylketone to the thiol group of Cys436, located in the ATP pocket of RSK2 CTKD^47^. Covalent modification irreversibly inhibited RSK2 C-terminal kinase activities^49^, inhibiting the N-terminal kinase activity. In contrast to these synthetic compounds, three natural compounds, SL0101, kaempferol and eriodictyol, have been suggested to inhibit RSK2 activity^48,50–52^. These natural compounds harbor flavonoid structures with a high degree of chemical similarity^53^. On the other hand, the mode of action studies of the compounds on RSK2 showed that the RSK2 activity was inhibited by SL0101 with an IC_50_ value of -89 nmol/L and kaempferol and eriodictyol with an IC_50_ value of -7 µmol/L of in test tube^53^. SL0101 and eriodictyol can also inhibit melanogenesis because kaempferol strongly inhibits melanogenesis. These results suggest that RSK2 targeting may be important for developing skin whitening and chemoprevention.

Canonically, signaling inducing RSK2 phosphorylation and activation is considered to be mediated via MEK-ERK MAPK signaling pathway. We also believe that MEK-ERK-RSK2 signaling pathway plays an important role in cell proliferation and cancer development^53^. On the other hand, previous our reports indicated that, while epidermal growth factor receptor-activated MAPK signaling pathway mainly transfers activation signaling via MEK-ERK-RSK2^48^, different growth factor, such basic fibroblast growth factor, differently activates RSK2 via direct interaction between bFGFR and RSK2^30^. Interestingly, decoupling between MAPK and PKA signaling pathways also is a well accepted concept when RAT-1, Swiss-3T3 and COS-7 cells were stimulated by EGF, forskolin, or LPA^54^. Moreover, our result demonstrated that LY294002, an PI3K inhibitor, dramatically increased AKT phosphorylation in contrast to our expectation (Fig. 2A). Moreover, U0126, an MEK inhibitor, also dramatically increased the ERK phosphorylation (Fig. 2B) in contrast our expectation. However, U0126 suppressed α-MSH-induced B16F10 cell darkness and melanogenesis (Fig. 2B,D) in contrast to LY294002, which increased synergistically with α-MSH in B16F10 cell darkness and melanogenesis (Fig. 2A,C). These results suggested that signaling pathways involved in melanogenesis is not operated via canonical signaling pathway. Interestingly, we found that, since RSK2 is a member of AGC kinase, RSK2 phosphorylates RxRxxS/T or RxxS/T motifs, which can be phosphorylated by AKT^24,48^. These results suggested to lead the experiment to confirm whether RSK2 can phosphorylate FOXOs or not. Based on this rationale, we searched the RSK2-mediated phosphorylation target motifs in the family members of FOXO (Fig. 4A). We further proved evidence in this study that RSK2-mediated FOXO4 phosphorylation regulated FOXO4 transactivation activity and melanogenesis. The involvement of FOXOs in melanogenesis was recently elucidated in a study indicating that FOXO3/FOXO6 phosphorylated by AKT inhibits melanogenesis^17,18^. In addition to this inhibitory mechanism, this study elucidated that FOXO4 phosphorylated by RSK2 increases melanogenesis. Nevertheless, it is unclear how α-MSH can activate RSK2. More importantly, the utilization of RSK2 inhibitors, such as kaempferol, eriodictyol, or SL0101, as whitening agents may have the advantage of inhibiting skin carcinogenesis induced by UV because RSK2 inhibition abrogated EGF- or TPA-induced cell transformation. In the future, the signaling pathway studies based on molecular target regulation will reveal the efficacy of these compounds in skin whitening.

## Methods

### Reagents and antibodies

Dulbecco’s Modified Eagle Medium (DMEM, Cat#: 10-013-CVR) and fetal bovine serum (FBS, Cat#: 26140-079) for cell culture were purchased from Corning (NJ, NY, USA). Dimethyl sulfoxide (DMSO, Cat#: 67-68-5) was purchased from Duchefa Farma B.V. (Haarlem, NH, Netherlands) for compound dilution and cell treatment. Fargesin (purity > 98% by high-performance liquid chromatography, Cat#: F1188) was purchased from Tokyo Chemical Industry (Tokyo, Japan). Kaempferol (purity > 98% by high-performance liquid chromatography, Cat#BD-D1339) was purchased from Biofron (La Mirada, CA, USA). Aschantin (purity > 99% by high-performance liquid chromatography) was provided by Dr. Dr S.-R Oh (Korean Research Institute of Bioscience and Biotechnology). U0126 (Cat#: S1102), BI-D1870 (Cat#: S2843), and LY294002 (Cat#: S1105) were obtained from Selleck Chemicals (Houston, TX, USA). Melanin (Cat#: M8631) was procured from Sigma-Aldrich (Saint Louis, MO, USA). Alpha-melanocyte stimulating hormone (α-MSH, Cat#: M0939, obtained from Sigma-Aldrich) was dissolved in ultra-pure water (Cat#: SM-Wo1-100, Geneall, Seoul, South Korea) and stored at -20 ℃ as a stock solution (1 mM). Cell signaling Technology supplied the following antibodies: phospho-p44/42 MAPK (ERK1/2, Cat#: 4370 and 9102, respectively), -p90RSK (Thr359/Ser363, Cat#: 9344), -AKT-Thr308 (Cat#: 2965), -AKT-Ser473 (Cat#: 4060), and -AKT substrates (recognize the phosphorylated RxxS/T motif, Cat#: 9614; recognized the phosphorylated RxRxxS/T motif, Cat#: 10,001), and total-p44/42 MAPK (ERK/12, Cat#: 9102), -AKT (pan, Cat#: 2920), and -FOXO1 (Cat#: 2880) antibodies. Antibodies including total-RSK2 (Cat#: SC-9986), -AFX1 (FOXO4, Cat#: SC-373877), -MITF (Cat#: SC-56725), -GST (Cat#: SC-138), and -β-actin (Cat#: SC-47778) were purchased from Santa Cruz Biotechnology (Santa Cruz, CA, USA). Antibodies that recognize tags, including Anti-HA-tag pAb-HRP-Direct (Cat#: M180-7), and Anti-DDDDK-tag pAb-HRP-Direct (Cat#: M185-7), were purchased from MBL Life Science (Woburn, MA, USA).

### Cell culture

B16F10 mouse melanoma cells, RSK2^+/+^ and RSK2^-/-^ MEFs, and HEK293T cells, which were purchased from the American type culture collection (Manassas, VA, USA), were cultured in Dulbecco’s Modified Eagle Medium (DMEM) supplemented with 10% FBS at 37 ℃, 5% CO_2_ incubator. B16F10 and RSK2^+/+^ and RSK2^-/-^ MEF cells with passage number 10 or less were used, and HEK293T cells with passage number 20 or less were used. The cells were passaged when the density reached approximately 90% confluence, and the medium was changed every other day.

### Establishment of stable cells

Viral particles were produced by the transfection of pLKO.1-sh-RSK2 or pCDH-CMV-MCS-EF1α-Puro-RSK2-Y707A and packaging vectors, psPAX2 and pMD2.G (obtained from Addgene), in HEK293T cells and harvested by filtration of the medium at 48 h using a 0.45 µm cellulose acetate syringe filter (Cat#: S6555, Sartorius, Göttingen, Germany). The viral infection was carried out by scattering the harvested medium onto the B16F10 cells with 2 μg/ml of polybren. The infected B16F10 cells were selected by culturing the cell with complete medium containing 1.5 µg/ml of puromycin (Cat#: A1113802, Thermo Fisher Scientific, Waltham, MA, USA) for two days.

### Chemical treatment

The chemical compounds used in this study were prepared in 1000 × stock solutions in DMSO and stored at -70 ℃ until used. The chemicals in the working solution were prepared by freshly diluted them in DMSO from the stock solution. The concentration of DMSO did not exceed 0.1% of final concentration in a complete cell culture medium. For signaling analysis, the cells were pretreated with chemical inhibitors for 30 min before α-MSH stimulation. The effects of chemicals on melanogenesis were observed at 36 h after treatment.

### UV irradiation

The effects of melanogenesis by UVA or UVB were examined by irradiating B16F10 melanoma cells with the indicated doses of UVA (7 mJ/cm^2^) or UVB (1 mJ/cm^2^) in a UV irradiation chamber (UV Messtechnik opsytec Dr. Gröbel GmbH, Ettlingen, Germany). UV absorption was maximized by rinsing the B16F10 cells with 1 × Ca^2+^/Mg^2+^-PBS before UV irradiation, overlaying them with a 1/2 media volume of 1 × Ca^2+^/Mg^2+^-PBS, and then irradiating them with UV light. After UV irradiation, the cells were replaced with complete culture medium and cultured in a 5% CO_2_ incubator for 36 h.

### Western blot

The cell lysates (20–30 µg) extracted from which B16F10 cells were treated with combination of UVA, chemicals, and α-MSH were used to analyze the total- and phospho-protein levels. The cell lysate extracted using RIPA cell lysis buffer [150 mM Sodium Chloride, 1% triton X-100, 1% sodium deoxycholate, 0.1% SDS, 50 mM Tris–HCl, 2 mM EDTA pH 7.5, (Cat#: R41000-010, GenDEPOT, Katy, TX, USA)], was dissolved by SDS-PAGE and transferred onto a polyvinylidene difluoride membrane (PVDF, Cat#: 88518, Merck Millipore, Burlington, MA, USA). The membranes were blocked with 5% milk blocking buffer for 1 h and hybridized with a specific primary and HRP-conjugated secondary antibodies as indicated. The level of total- and phospho-proteins was visualized using an enhanced chemiluminescence detection system with an Immobilon Forte Western HRP substrate (Cat#: WBLUF0500, obtained from Merck Millipore). β-actin was used as an internal control for equal protein loading.

### Melanin content assay

B16F10 cells treated with a combination of UVA, UVB, chemical compounds, or α-MSH were harvested, washed with 1 × ice-cold PBS, and lysed by adding 10% DMSO in 1 N NaOH. Melanin solubility was enhanced by sonicating the cell lysates for 15 min with 30 s of the sonication/resting cycle and heated at 80 ℃ for 1 h. The cleared cell lysates obtained by centrifugation were used to determine the melanin content by spectrophotometry (OD^490^ nm) using an xMark™ Microplate Absorbance spectrophotometer (Bio-Rad, Hercules, CA, USA). The absolute melanin content (ng/mg protein) was converted by comparing the melanin standard curve and compensating for it with the protein amount in the cleared cell lysates.

### Construction of expression vectors

The expression vectors for FOXO genes, including pCS2 + N-Flag-FOXO1A (Cat#: 153141), pCS2 + N-Flag-FOXO3 (Cat#: 153142), and pCS2 + N-Flag-FOXO4 (Cat#: 153143), were purchased from Addgene (Watertown, MA, USA). Supplemental Fig. S1 lists the primer sequences for the expression vector construction in this study.

### Immunoprecipitaion

The cell lysates (200 µg) extracted from HEK293T cells were used to validate the protein–protein interaction between RSK2 and FOXOs by immunoprecipitation (IP). Briefly, the HEK293T cells transfected with indicated expression vectors were lysed with an NP-40 cell lysis buffer [50 mM Tris–HCl (pH 8.0), 120 mM NaCl, and 0.5% NP-40] by freezing and thawing. The cell lysates containing equal amounts of the proteins were coupled with HA-specific antibody conjugated bead (HA-bead) (Cat#: A2095, Sigma-Aldrich) overnight at 4 ℃. The HA-beads were collected, washed with NETN wash buffer [20 mM Tris (pH 8.0), 100 mM NaCl, 1 mM EDTA, and 0.5% NP-40], resuspended with 35 µl of 1 × SDS sample buffer, and boiled at 95 ℃ for 5 min. RSK2 and FOXOs interaction was visualized by Western blotting using specific antibodies as described in Materials and Methods.

### Partial purification of GST-fusion proteins

For in vitro kinase assay and pull down assay, glutathione S-transferase (GST)-fused FOXO4 and RSK2 expression vectors were introduced in BL21 by induction with IPTG (final concentration 0.1 mM) for 2 h at 30 ℃ or with IPTG (final concentration 0.5 mM) for 2 h at 16 ℃. The BL21 cells were harvested, re-suspended with ice-cold IBW buffer [20 mM Tris–HCl, 10 mM EDTA, and 1% Triton X-100], and retained for 30 min on ice with pipetting every 5 min. The crude cell supernatant collected by centrifugation was coupled with a glutathione separopore bead (Bioworld, OH, USA, Cat#: 20,181,050–2) and washed with the IBW buffer. The GST-tagged FOXO4 or -RSK2 proteins were obtained by adding 60 μl of 10 mM reduced glutathione or used directly for the pulldown assay. The interaction of RSK2 and FOXO-1 or -4 were confirmed and visualized by ex vivo pulldown/Western blotting using Flag-specific antibodies.

### In vitro kinase assay

The bead-bound GST-FOXO4 protein expressed in BL21 was combined with commercially active RSK2 with cold ATP (final concentration 80 μM) and conducted kinase reaction at 30 ℃ for 30 min. The reaction mixtures were combined with the 6 × sample buffer, boiled at 95 ℃ for 5 min, and resolved in SDS-PAGE. The phosphorylated FOXO4 proteins were visualized by Western blotting using RxRxxS/T- or RxxS/T-specific antibodies, as described above.

### Transactivation activity analysis (Luciferase assay)

The B16F10 melanoma cells or RSK2^+/+^ and RSK2^-/-^ MEF cells were co-transfected pBIND-FOXO4 vectors and pG5-luciferase in a 12-well cell culture plate. The cells cultured for 24 h in complete medium were added the 150 µl of luciferase cell lysis buffer (Cat #: J3081, Promega, Madison, WI, USA). The cell cell lysates (100 µl) were used to measure firefly luciferase activity using a VICOTR X3 multilabel plate reader (PerkinElmer, MA, USA). The firefly luciferase activities were compensated for equal transfection with the *Renilla* luciferase activity. The relative fold changes of luciferase activity were compared to the mock control group.

### Cytotoxicity assay

The cytotoxicity for kaempferol was evaluated using an MTS assay kit in B16F10 melanoma cells. Briefly, B16F10 cells (1.5 × 10^4^ /well) were seeded into 96-well plates, cultured overnight, and treated with the indicated doses of kaempferol for 36 h. The MTS assay (Cat#: G1111, obtained from Promega) was conducted to measure the cell viability at 24 h and 36 h. The relative viability was obtained by a comparison with the non-treated control group.

### Statistical analysis

Statistical analysis was performed using three independent experiments or a triplicate experiment. Statistical analysis was performed using Microsoft Excel. The data are presented as the mean ± SEM. The statistical significance was obtained using student *t*-tests between the two groups. P values < 0.05 (two-tailed) were considered significant.

### Supplementary Information

Supplementary Figures.

Supplementary Information.

## Data Availability

All data generated or analyzed during this study are included in this published article (and its Supplementary Information files). The plasmids are available from the corresponding author, Yong-Yeon Cho, upon request.
