# Supplementary material for: Ribosomal S6 kinase 2-forkhead box protein O4 signaling pathway plays an essential role in melanogenesis
Source: Sci Rep. 2024 Apr 24;14:9440. doi: 10.1038/s41598-024-60165-9 (PMC11043394; doi:10.1038/s41598-024-60165-9)

Whole blots Fig. 1 by DH

Fig 1A

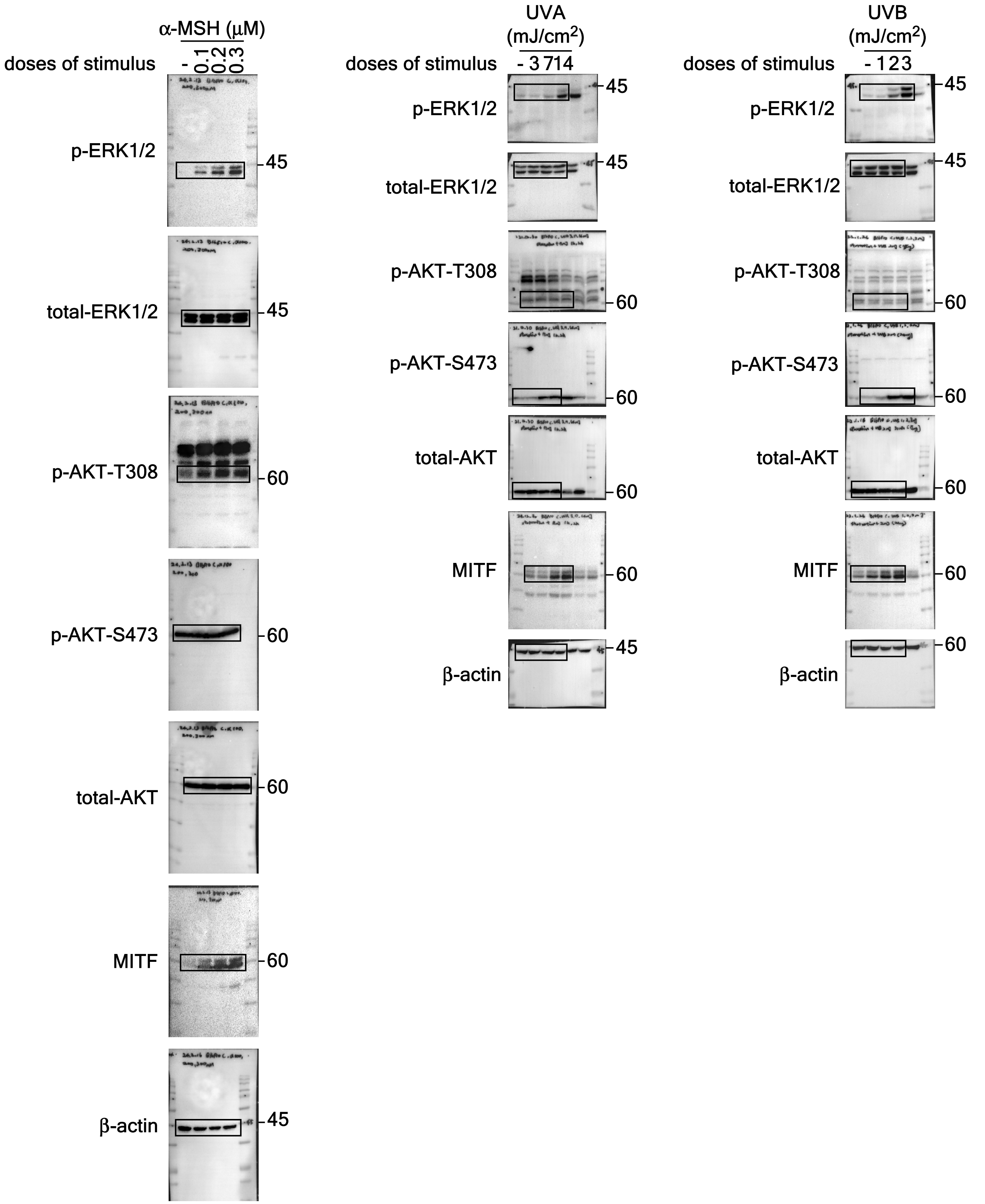

Whole blots Fig. 2 by DH

Fig 2A

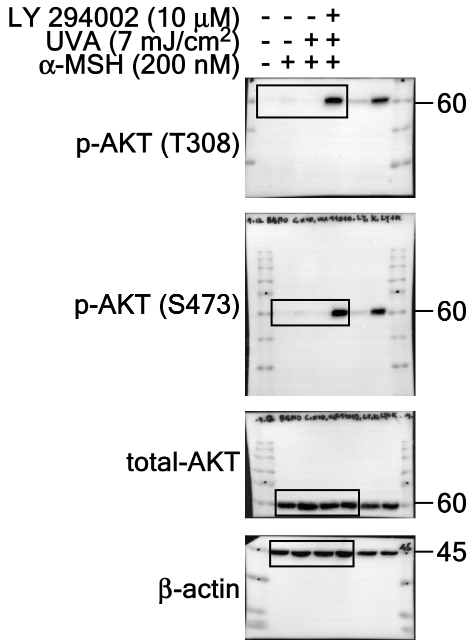

Fig 2B

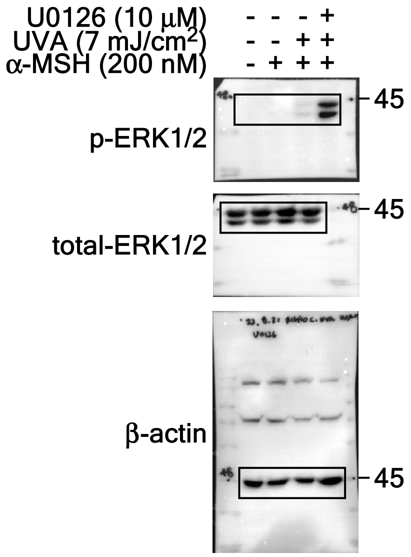

Whole blots Fig. 3 by DH

Fig 3A

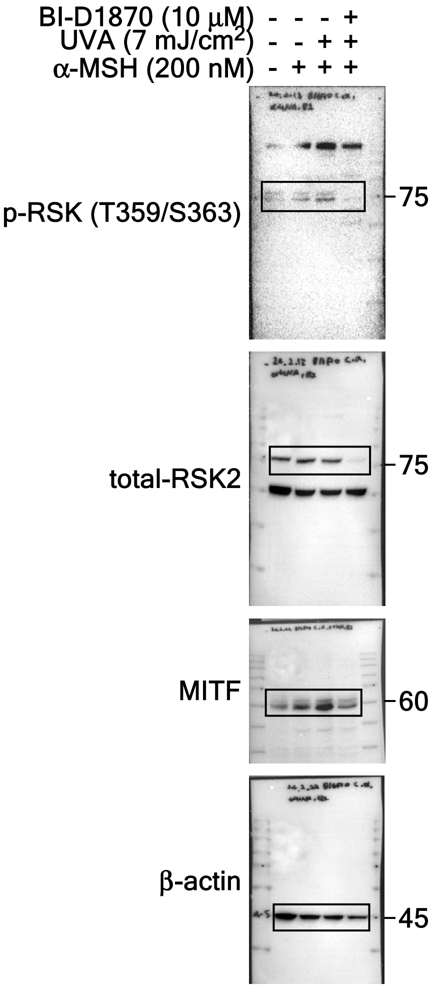

Fig 3C

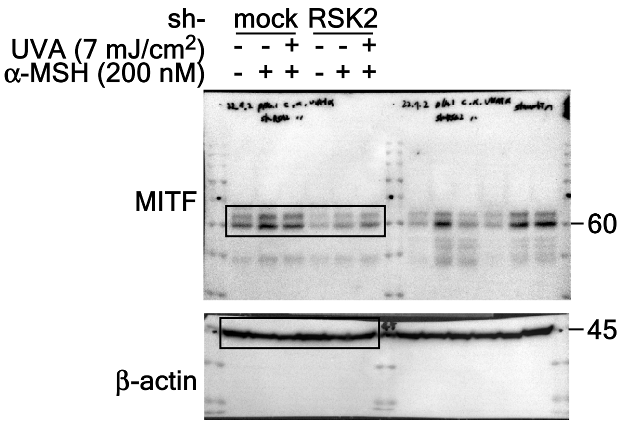

Fig 3E

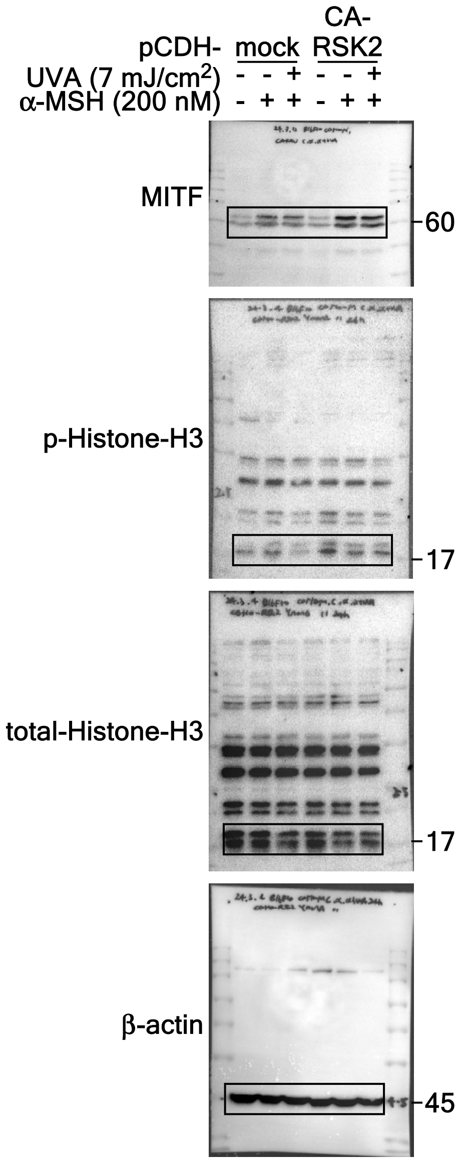

Whole blots Fig. 4 by DH

Fig 4B

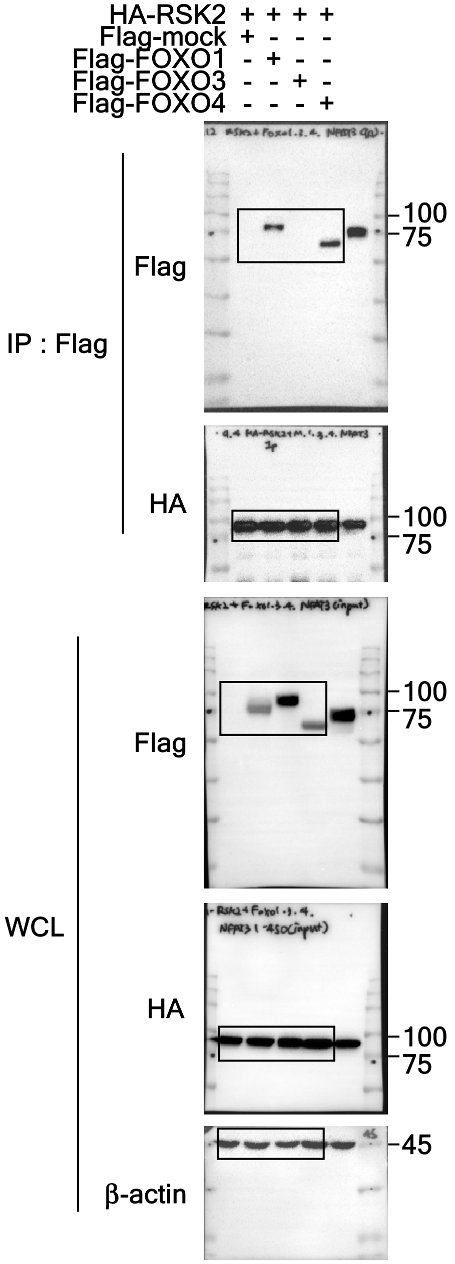

Fig 4C

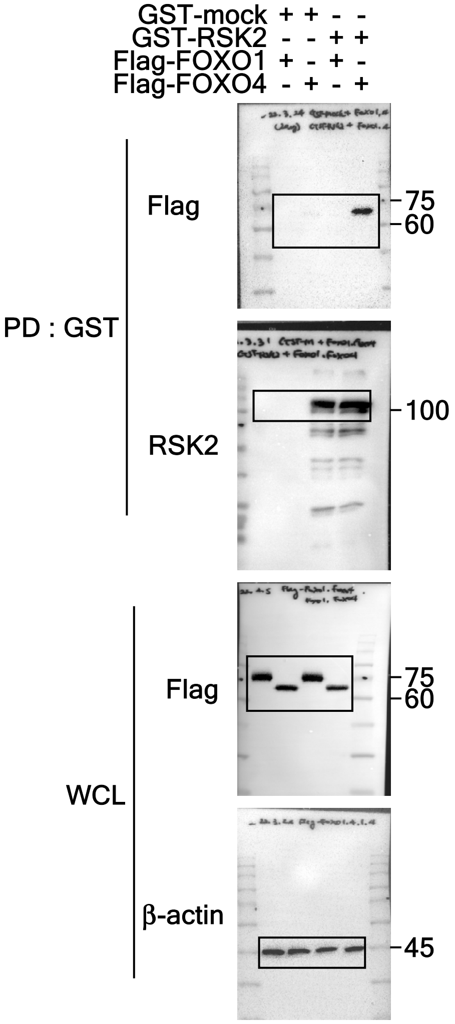

Fig 4D

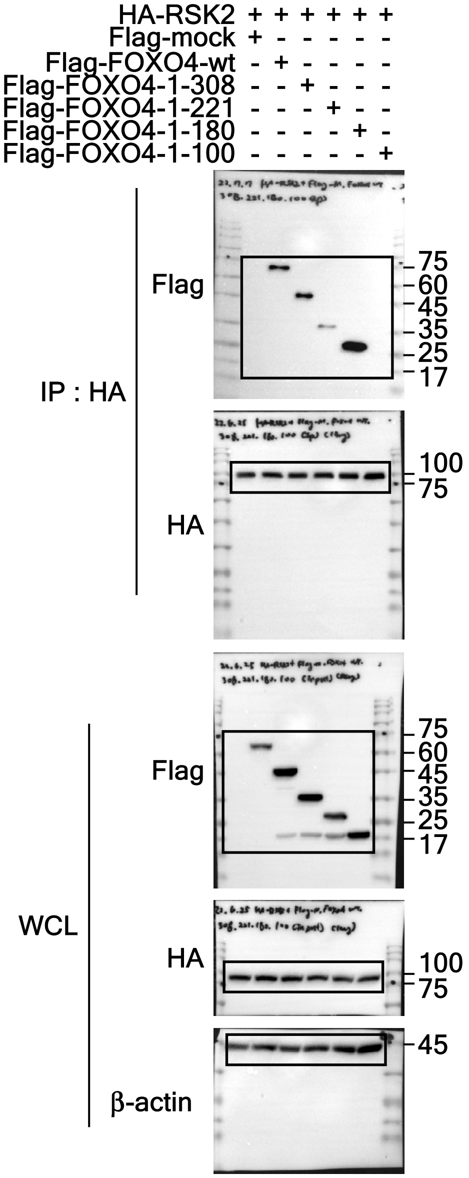

Whole blots Fig. 5 by DH

Fig 5B

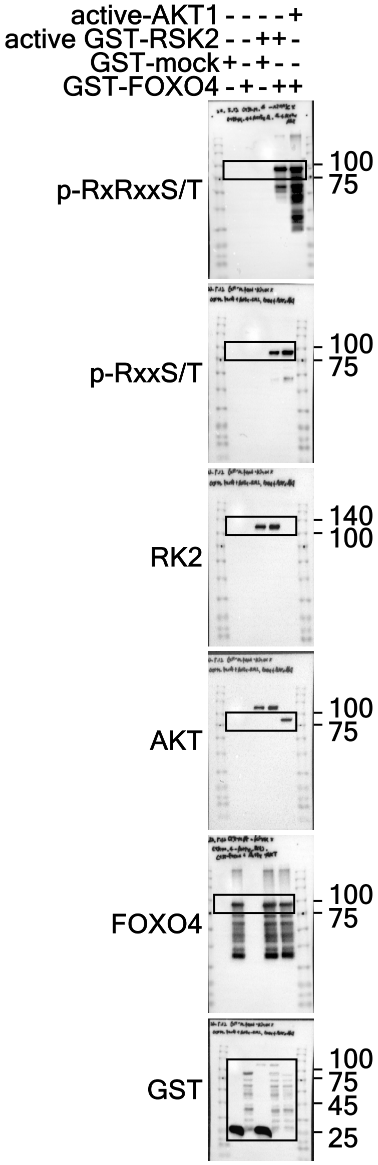

Fig 5C

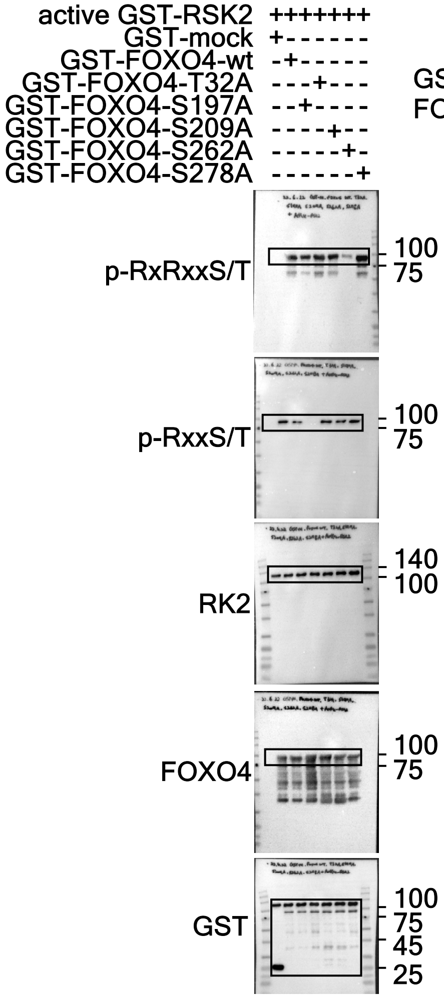

Fig 5D

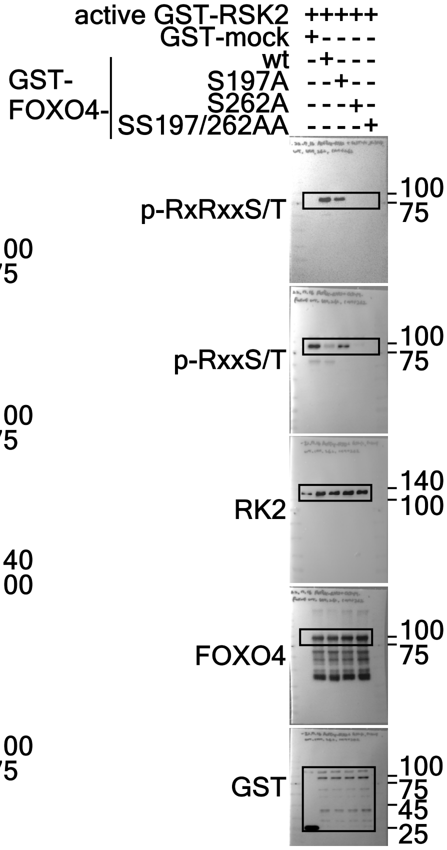

Fig 5E

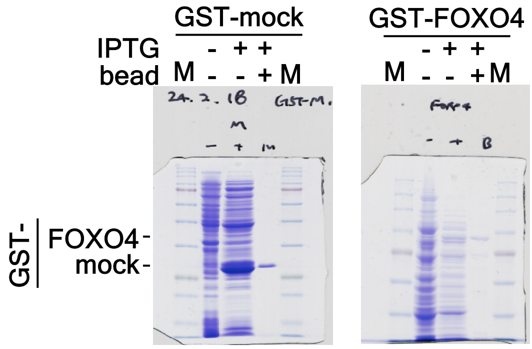

Fig 5F

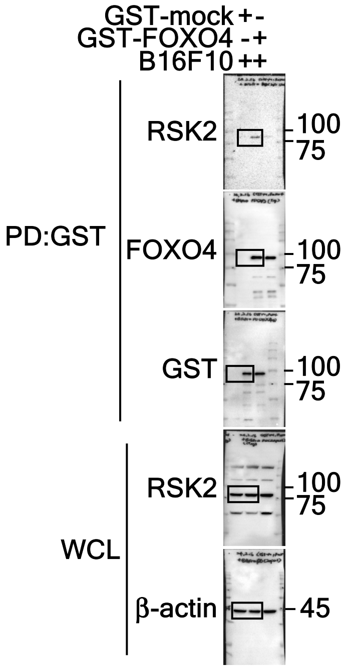

Whole blots Fig. 6 by DH

Fig. 6H

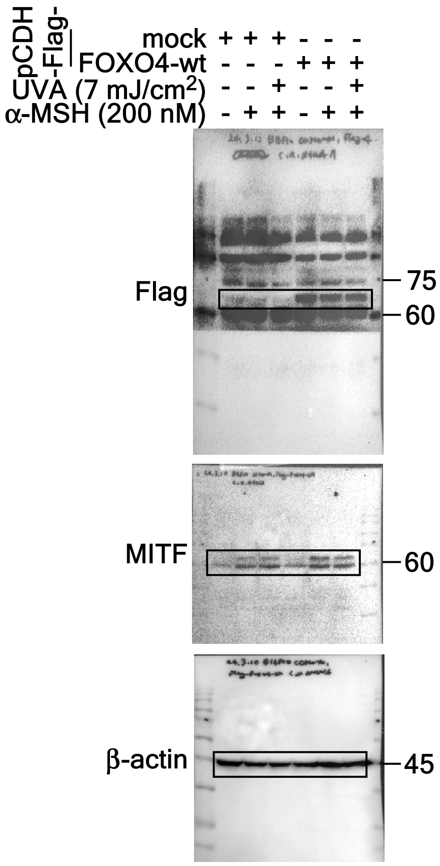

Whole blot-Supplementary Fig. 1 by DH

Suppl. Fig. 1

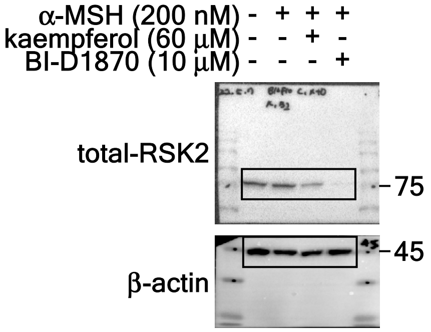

Whole blot-Supplementary Fig. 2 by DH

Supp. Fig. 2B

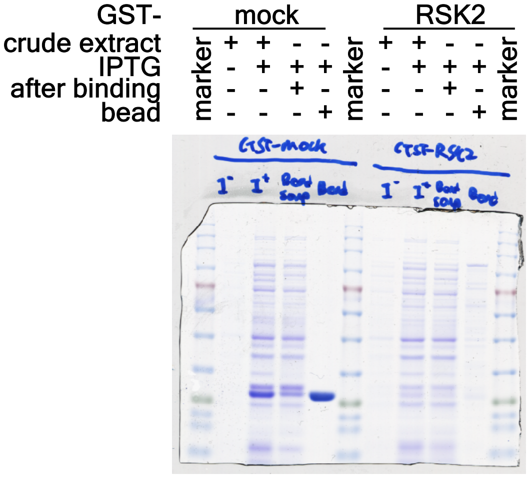

Whole blot-Supplementary Fig. 3 by DH

Supp. Fig. 3A

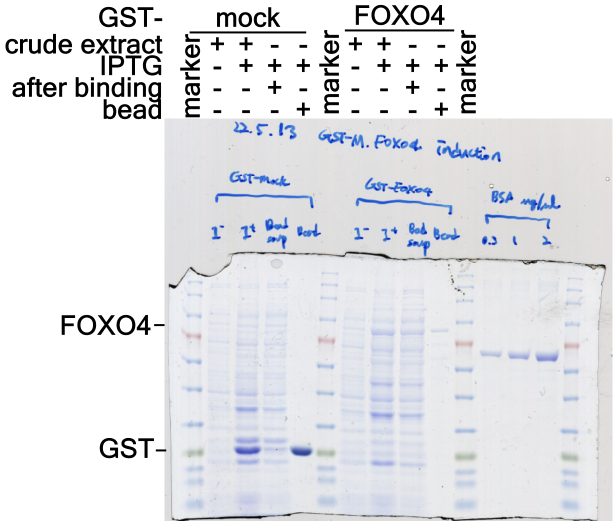

Supp. Fig. 3C

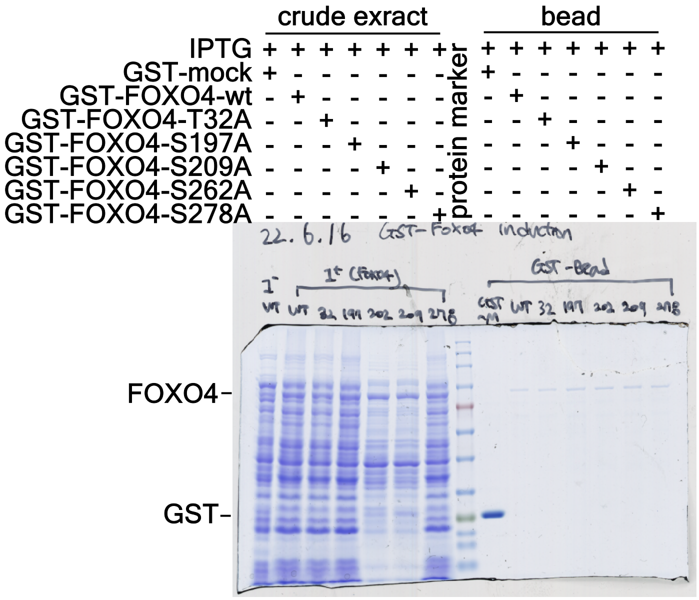

Supp. Fig. 3D

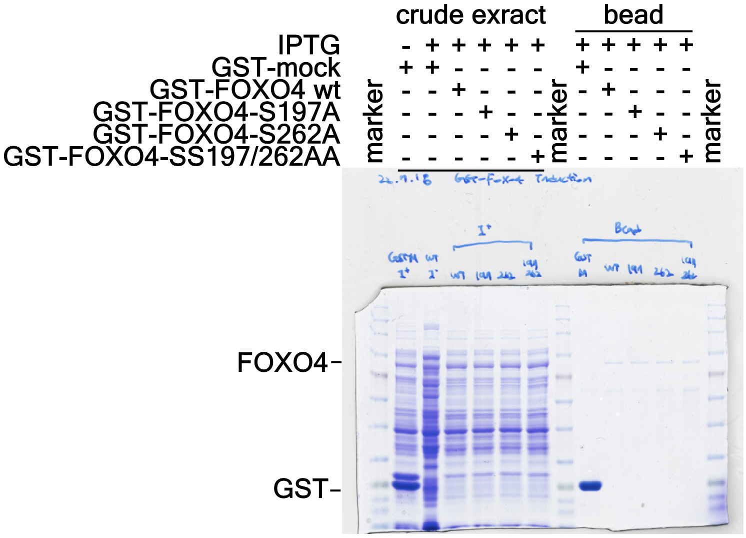

Supplement: Supplementary file 1 — Supplementary Figures. [file 41598_2024_60165_MOESM1_ESM.pdf]
