# Supplementary material for: Ribosomal S6 kinase 2-forkhead box protein O4 signaling pathway plays an essential role in melanogenesis
Source: Sci Rep. 2024 Apr 24;14:9440. doi: 10.1038/s41598-024-60165-9 (PMC11043394; doi:10.1038/s41598-024-60165-9)
Supplement: Supplementary file 2 — Supplementary Information. [file 41598_2024_60165_MOESM2_ESM.pdf]

[Supplementary Information]

RSK2-FOXO4 signaling pathway plays an essential role in melanogenesis

Dohyun Jeung[1], Ga-Eun Lee[1], Weidong Chen[1], Jiin Byun[1], Soo-Bin Nam[1,2], You-Min Park[1], Hye Suk Lee[1], Han Chang Kang[3], Joo Young Lee[1], Kwang Dong Kim[4], Young-Soo Hong[5], Cheol-Jung Lee[2], Dae Joon Kim[6], and Yong-Yeon Cho[1,*]

Contents:

1. 3 Supplementary tables
2. 4 Supplementary Figures

Supplementary Tables: 3 tables

Supplementary Table 1. Primer lists for expression vector construction

Vector name	Forward/ Reverse	Sequence	Restriction enzyme
pCDH-CMV-MCS-EF1α-Puro-RSK2-Y707A	F	5'-ATTTAAATATGCCGCTGGCGCAGCTGGCGGACCCG-3'	*Swa* I
	R	5'-TTGCGGCCGCTCACAGGGCTGTTGAGGTGATTTTT-3'	*Not* I
pBIND-FOXO4	F	5'-GCGTCGACTTATGGATCCGGGGAATGAG-3'	*Sal* I
	R	5'-TTGCGGCCGCTCAGGGATCTGGCTCAAA-3'	*Not* I
pGEX-5X-1-FOXO4	F	5'-GCGTCGACTCATGGATCCGGGGAATGAG-3'	*Sal* I
	R	5'-TTGCGGCCGCTCAGGGATCTGGCTCAAA-3'	*Not* I

Supplementary Table 2. Primer lists for FOXO4 serial deletion mutant construction

Vectors	Forward/ Reverse	Sequence
pGEX-5X-1-FOXO4-serial deletion	F	5'-AGTCATGCCTGCTCGAGCCTCTAGAACTATA-3'
pGEX-5X-1-FOXO4-1-308	R	5'-ATCTAACAGCTCTAGACCTTCATTGAGGGTG-3'
pGEX-5X-1-FOXO4-1-221	R	5'-AGCTGGCAGCAGAGATGGTTTCTTGGGG-3'
pGEX-5X-1-FOXO4-1-180	R	5'-CAGCATCCAAGCGATTTTGCCGGTGGCC-3'
pGEX-5X-1-FOXO4-1-100	R	5'-GGCATTCCGGAGGGAGCCTCCCTTCCGAGGA-3'

Supplementary Table 3. Primer lists for FOXO4 single- and double-point mutant construction

Vectors	Forward/ Reverse	Sequence
pGEX-5X-1-FOXO4-T32A	F	5’-CCCCGCTCCTGCGCATGGCCCCTT-3’
	R	5’-AAGGGGCCATGCGCAGGAGCGGGG-3’
pGEX-5X-1-FOXO4-T197A	F	5’-CGGGCCGCAATGGATAGCAGC-3’
	R	5’-GCTGCTATCCATTGCGGCCCG-3’
pGEX-5X-1-FOXO4-T209A	F	5’-GGCATTCCGGAGGGAGCCTCCCTTCCGAGGA-3’
	R	5’-CTTCTTGGGGGCTTTTGCGCGGCCCCGGAGCAG-3’
pGEX-5X-1-FOXO4-T262A	F	5’-CCACGAAGCAGTGCAAATGCCAGCAGT-3’
	R	5’-ACTGCTGGCATTTGCATGCTTCGTAA-3’
pGEX-5X-1-FOXO4-T278A	F	5’-CCCTTGAGGCCAGAGGCAGAGGTGCTGGCGGAG-3’
	R	5’-CTCCGCCAGCACCTCTGCCTCTGGCCTCAAGGG-3’

[Supplementary Figure Legends]

Supplementary Fig. 1 by DH

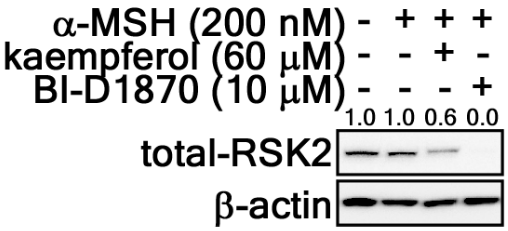

Supplementary Figure 1. RSK2 activity inhibition supresses RSK2 protein levels. Chemical inhibitiors, such as kaempferol and BI-D1870, of RSK2 activity were co-treated with α-MSH (200 nM) in B16F10 melanoma cells for 36 h. The cell lysates from the cells were used to visualize the total protein levels of RSK2 by Western blotting. β-actin was used as an internal control to verify the equal protein loading.

Supplementary Fig. 2 A+B+C by DH

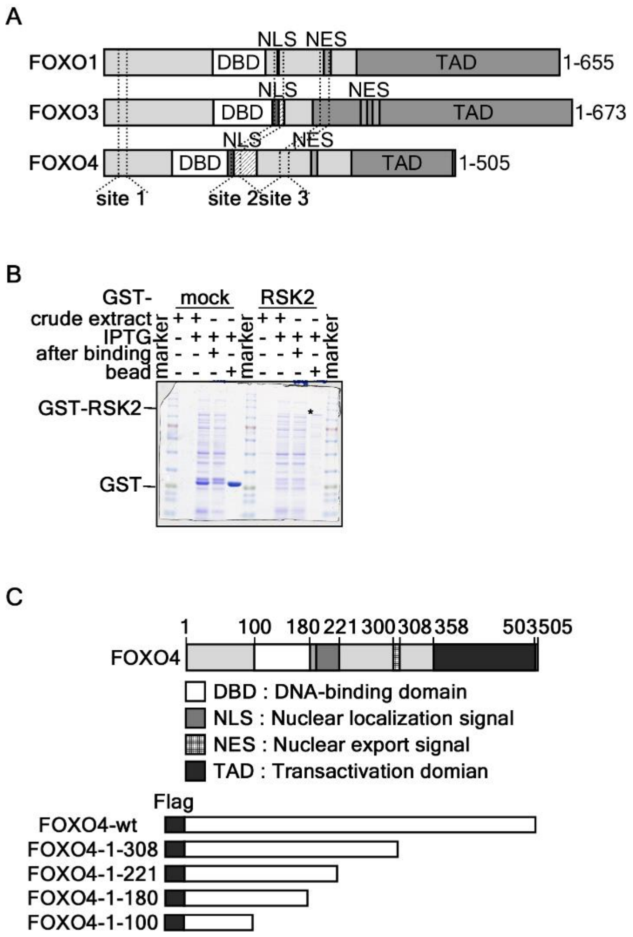

Supplementary Figure 2. (**A**) Schematic structures of FOXO 1, 3, and 4. DBD, DNA binding domain; NLS, nuclear localization sequence; NES, nuclear exporting signal; and TAD, transactivation domain. Site 1, 2, and 3 indicate the location of potent AKT and RSK2 target phospho-sensensus sequences in FOXO 1, 3, and 4 as indicated. (**B**) Confirmation of GST-RSK2 expression for *ex vivo* GST-RSK2 pull-down assay. BL21 E.coli transformed with pGEX-5X-1-RSK2-FL were used to confirm whether GST-RSK2 protein could be used for the pull-down assay, as described in Materials and Methods. The GST-separopore beads bound with GST-RSK2 proteins were electrophoresed and visualized by Coomassie brilliant blue R-250 staining. The GSR-RSK2 beads were used to determine the ex-vivo pull-down assay in **Fig. 4C**. (**C**) Construction of FOXO4 serial deletion mutants. Flag-tagged FOXO4 deletion mutants from the C-terminus were recombined into the pcDNA-Flag mammalian expression vector. The inframed FOXO4-wt, -1-308, -1-221, -1-180, and -1-100 vectors were confirmed by DNA sequencing. These vectors were used to determine the interaction domain of FOXO4 with RSK2-wt as shown in **Fig. 4D**.

Supplementary Fig. 3 A+B+C+D By DH

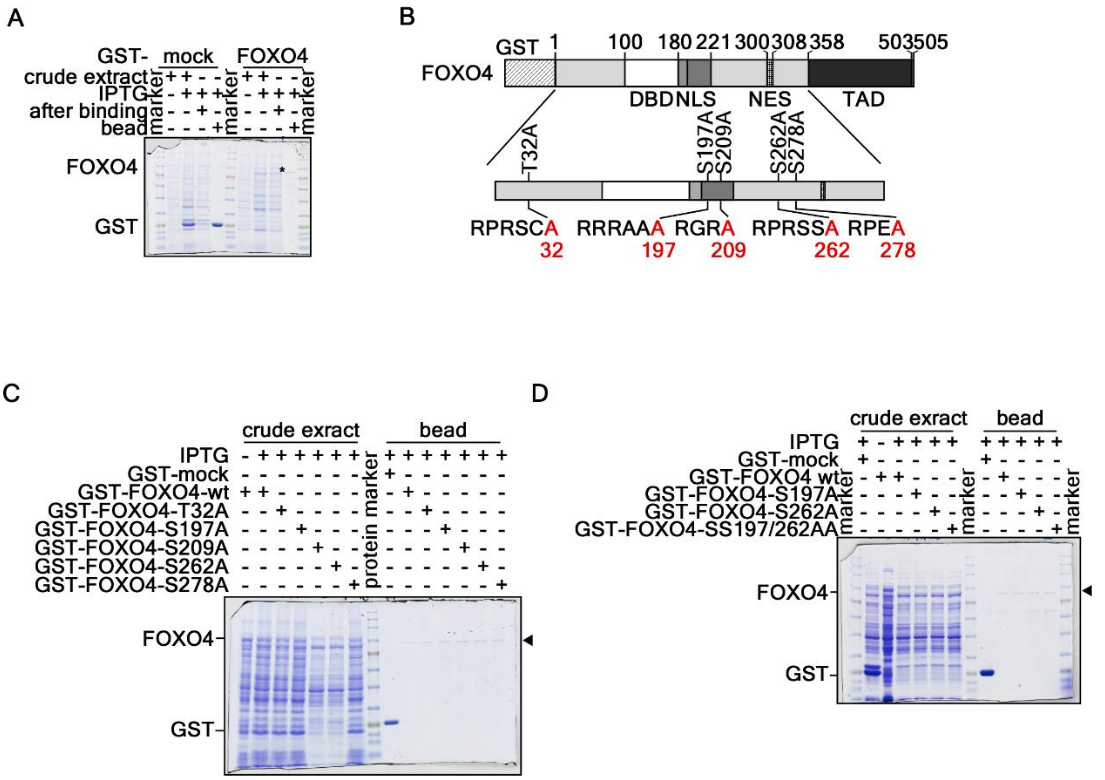

Supplementary Figure 3. (**A**) Partial purification of GST-FOXO4 proteins. BL21 lysates transformed with pGEX-5X-1-FOXO4 were used to purify the FOXO4 proteins partially. The bead-GST-FOXO4 proteins were visualized by Coomassie Brillian blue R-250 staining. Arrow, FOXO4 proteins. (**B**) Construction map of SGT-FOXO4-wt and -point mutants (GST-FOXO4-T32A, -S197A, -S209A, -S262A, and -S278A) as indicated. (**C**) Bacterial cell lysates transformed with each of GST-FOXO4-wt and point mutants (GST-FOXO4-T32A, -S197A, -S209A, -S262A, and -S278A) were used to bind GST-beads. The GST-FOXO4-wt and -mutants bound GST-bead were obtained and confirmed the quality to be used for in vitro kinase assay as shown in Fig. 5C. The proteins were visualized by Coomassie Brillian blue R-250 staining. Arrow head, FOXO4 proteins. (**D**) Bacterial cell lysates were transformed with GST-FOXO4-wt, -S197A, -S262A, and S197A/S262A mutants to bind with the GST-bead. The GST-FOXO4-wt and -mutant bound GST-bead were obtained and confirmed the quality to be used for the *in vitro* kinase assay, as shown in **Fig. 5D**. The proteins were visualized by Coomassie Brillian blue R-250 staining. Arrow, FOXO4 proteins.

Supplementary Fig. 4 by DH

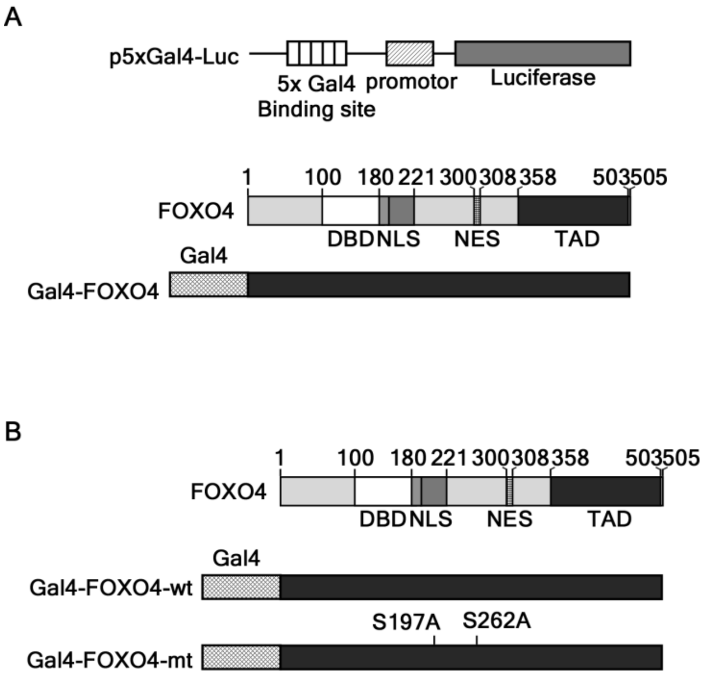

Supplementary Figure 4. Construction maps of FOXO4 transactivation activity. Gal4 DNA binding domain was fused with FOXO4 at the N-terminal end. To measure the transactivation activity of FOXO4, the Gal4 binding consensus sequence in pG5-luciferase was recombinded with a minimal promoter-luciferase vector. The plasmids were confirmed by DNA sequencing.
